# Non-coding RNA yREX3 from human extracellular vesicles exerts macrophage-mediated cardioprotection via a novel gene-methylating mechanism

**DOI:** 10.1093/eurheartj/ehae357

**Published:** 2024-06-12

**Authors:** Alessandra Ciullo, Liang Li, Chang Li, Kara Tsi, Colin Farrell, Matteo Pellegrini, Eduardo Marbán, Ahmed G E Ibrahim

**Affiliations:** Cedars-Sinai Medical Center, Smidt Heart Institute, 8700 Beverly Blvd, 1090 Davis Bldg, Los Angeles, CA 90048, USA; Cedars-Sinai Medical Center, Smidt Heart Institute, 8700 Beverly Blvd, 1090 Davis Bldg, Los Angeles, CA 90048, USA; Cedars-Sinai Medical Center, Smidt Heart Institute, 8700 Beverly Blvd, 1090 Davis Bldg, Los Angeles, CA 90048, USA; Cedars-Sinai Medical Center, Smidt Heart Institute, 8700 Beverly Blvd, 1090 Davis Bldg, Los Angeles, CA 90048, USA; Department of Molecular, Cell, and Developmental Biology, University of California, Los Angeles, Los Angeles, CA, USA; Department of Molecular, Cell, and Developmental Biology, University of California, Los Angeles, Los Angeles, CA, USA; Cedars-Sinai Medical Center, Smidt Heart Institute, 8700 Beverly Blvd, 1090 Davis Bldg, Los Angeles, CA 90048, USA; Cedars-Sinai Medical Center, Smidt Heart Institute, 8700 Beverly Blvd, 1090 Davis Bldg, Los Angeles, CA 90048, USA

**Keywords:** Myocardial infarction, Macrophages, Small non-coding RNA, Pick1, Efferocytosis, Inflammation

## Abstract

**Background and Aims:**

Extracellular vesicles (EVs) secreted by cardiosphere-derived cells exert immunomodulatory effects through the transmission of small non-coding RNAs.

**Methods:**

The mechanism and role of yREX3, a small Y RNA abundant in EVs in myocardial injury, was investigated.

**Results:**

yREX3 attenuates cardiac ischaemic injury by selective DNA methylation. Synthetic yREX3 encapsulated in lipid nanoparticles triggers broad transcriptomic changes in macrophages, localizes to the nucleus, and mediates epigenetic silencing of protein interacting with C kinase-1 (Pick1) through methylation of upstream CpG sites. Moreover, yREX3 interacts with polypyrimidine tract binding protein 3 (PTBP3) to methylate the Pick1 gene locus in a DNA methyltransferase–dependent manner. Suppression of Pick1 in macrophages potentiates Smad3 signalling and enhances efferocytosis, minimizing heart necrosis in rats with myocardial infarction. Adoptive transfer of Pick1-deficient macrophages recapitulates the cardioprotective effects of yREX3 *in vivo*.

**Conclusions:**

These findings highlight the role of a small Y RNA mined from EVs with a novel gene-methylating mechanism.

## Structured Graphical Abstract

**Structured Graphical Abstract:**
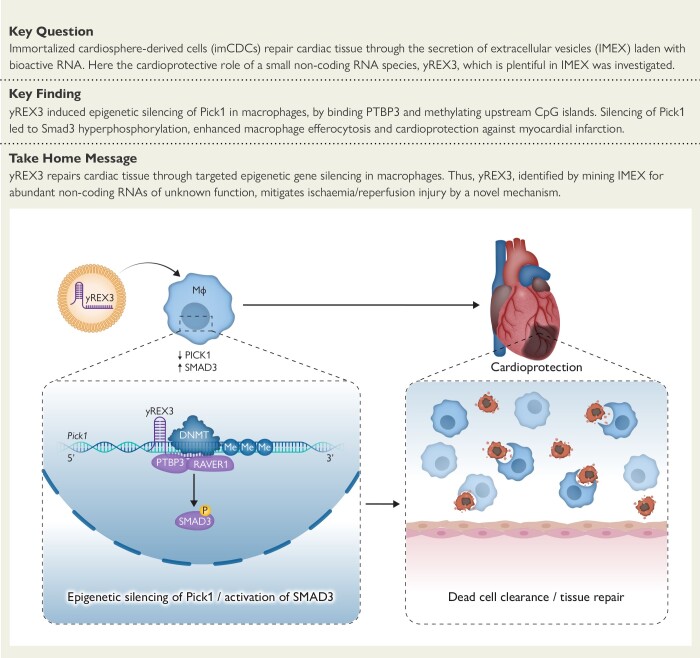
Small non-coding RNA, yREX3, induces methylation-mediated silencing of Pick1. Pick1 silencing in macrophages enhances their capacity to clear dead cells and promote tissue healing after injury.

**See the editorial comment for this article ‘RNAand the emerging potential of bio-inspired molecules in cardiovascular disease therapies’, by N. Ahmed and D.R. Davis, https://doi.org/10.1093/eurheartj/ehae348.**

**Translational perspective**

Although percutaneous coronary intervention has revolutionized the management of acute myocardial infarction, overall outcomes remain suboptimal. Adjunctive cardioprotective drugs represent one possible therapeutic option, but such drugs would need to be effective after reperfusion to be clinically viable. By mining extracellular vesicles (EVs) that are known to be cardioprotective, yREX3, a plentiful non-coding RNA of unknown function was investigated. Synthetic yREX3, loaded into lipid nanoparticles, altered gene expression and enhanced the phagocytic function of macrophages. In a rodent model of ischaemia/reperfusion, yREX3 limited infarct size even when given after reflow. The mechanism of action, via suppression of Pick1 in macrophages, is unprecedented. These findings establish yREX3 as a novel cardioprotective agent worthy of further translation. More generally, the discovery paradigm for yREX3—mining therapeutic EVs for plentiful RNAs with disease-modifying bioactivity—stands to identify other promising therapeutic candidates.

## Introduction

The clinical management of myocardial infarction (MI) has been transformed by percutaneous coronary intervention (PCI). Other than reperfusion, however, nothing has been shown convincingly to decrease infarct size, despite seven decades of investigation.^1^ Extracellular vesicles (EVs) have recently come to the fore as a new category of cardioprotective agents that may, at least in principle, work in a manner adjunctive to PCI.^2,3^ We became interested in EVs, as they mediate the disease-modifying bioactivity of cardiosphere-derived cell (CDC) therapy for MI^3–5^ and Duchenne muscular dystrophy.^6,7^ Extracellular vesicles produced by CDCs (CDC-EVs) contain >10 000 molecularly distinct non-coding RNA (ncRNA) entities with plausible but uncharacterized bioactivity.^8^ In a search for molecularly defined cardioprotective agents within EVs, we focus on RNA, as EV bioactivity is virtually eliminated when the contents are exposed to RNase, and on ncRNA, as intact messenger RNAs are relatively rare in CDC-EVs.^8^ Y RNAs are of interest because they are abundant in CDC-EVs (18% of small RNAs^8^) and poorly characterized. Y RNAs are highly conserved ncRNAs that bind to ribonucleoproteins La and Rho.^9^ In mammalian cells, Y RNAs are required for nuclear chromosomal replication,^10^ but their function is otherwise somewhat obscure. Functional studies on Y RNAs and their fragments found in EVs identified putative immunomodulatory effects.^9^ When manufactured synthetically and delivered *in vivo* in lipid nanoparticles (LNPs), one Y RNA exceptionally plentiful in CDC-EVs—EV-YF1—exerts therapeutic benefits in models of MI^8,11^ and cardiac hypertrophy.^12,13^ Likewise, EVs from immortalized CDCs (imCDCs),^14,15^ which are beneficial in models of MI,^14^ arrhythmogenic cardiomyopathy,^16^ and heart failure,^17^ richly express a small Y RNA, NT4,^15^ which is itself bioactive.^11^ Both EV-YF1 and NT4 are homologous to the 5′ region of the human Y RNA4 gene. In this study, we identify yREX3, a non-overlapping small Y RNA plentiful in immortalized CDC-EVs and find it to be profoundly cardioprotective in rats even when administered post-MI. Mechanistically, yREX3 acts via epigenetic modification of the Pick1 gene, which encodes a critical adaptor protein in macrophages. Thus, yREX3 is the lead compound for a new class of cardioprotective ncRNA drugs.

## Methods

### Animal models

#### Myocardial infarction, intracoronary delivery, and intravenous injection

Rats were housed in a pathogen-free facility (cage bedding: Sani-Chips, PJ Murphy) with a 14 h/10 h light/dark cycle with food [PicoLab Rodent Diet 20 (No. 5053), LabDiet] and water provided *ad libitum*. *In vivo*, experimental protocols were performed on 7- to 10-week-old female Wistar-Kyoto (Charles River Labs, Wilmington, MA, USA). To induce MI, a thoracotomy was performed at the fourth intercostal space to expose the heart under general anaesthesia. A 7-0 silk suture was then used to ligate the left anterior descending coronary artery, which was removed after 45 min to allow for reperfusion. Twenty minutes later, vehicle [phosphate-buffered saline (PBS) only], IMEX (1 × 10^10^ particles in 100 µL PBS), yREX3 (400 ng with DharmaFECT), or scrambled sequence (400 ng with DharmaFECT) were injected into the left ventricle (LV) cavity during aortic cross-clamp to achieve intracoronary delivery, for 20 s.^18^ For intravenous injection, yREX3 (400 ng with DharmaFECT in 50 µL) was injected in the retro-bulbar space.^19^

#### Adoptive transfer of macrophages

Twenty minutes after reperfusion, Mϕ^Veh^, Mϕ^yREX3^, Mϕ^Scr^, and Mϕ^siPick1^ (3 × 10^6^ cells/group in 300 µL of saline, generated as described above) were injected into the tail vein and animals sacrificed after 48 h to quantify infarct mass. In another set of experiments, Mϕ^Veh^ and Mϕ^yREX3^ were stained with the lipophilic dye DiO for 30 min at 37°C and then injected into the tail vein (3 × 10^6^ cells/group). Rats were sacrificed after 4 h for immunostaining and terminal deoxynucleotidyl transferase dUTP nick end labeling (TUNEL) assay.

### Infarct phenotyping

#### 2,3,5-Triphenyl-2H-tetrazolium chloride staining

Two days post-MI, 10% KCl was injected into the LV to arrest hearts in diastole. Then, hearts were harvested, washed in PBS, and cut into 1 mm sections from apex to base, above the infarct zone. Sections were incubated with 1% solution 2,3,5-triphenyl-2H-tetrazolium chloride (Sigma-Aldrich) for 30 min at 37°C in the dark and washed with PBS. Then, sections were imaged and weighed. The infarcted zones (white) were delineated from viable tissue (red) and analysed (ImageJ software). Infarct mass was calculated in the tissue sections according to the following formula: (infarct area/tot area)/weight (mg).

#### Cardiac Troponin I enzyme-linked immunosorbent assay

Blood was collected from animals at 24 h (from the tail vein) or at the study endpoint (from the heart) in ethylenediaminetetraacetic acid (EDTA) tubes. After being left undisturbed at 4°C for 30 min, plasma was obtained after 15 min centrifugation at 3220 *g*. Cardiac TnI was quantified using the Rat cardiac Troponin I enzyme-linked immunosorbent assay (ELISA) kit (Life Diagnostics), according to the manufacturer’s protocol.

### Assessing yREX3 abundance in peripheral blood mononuclear cells following intravenous injection in healthy rats

Peripheral blood mononuclear cells (PBMCs) were isolated from rat peripheral blood collected in EDTA-containing tubes and separated with Ficoll–Hypaque density gradients using the following protocol: 4 mL of blood/animal was diluted by adding two volumes of sterile PBS. In a 50 mL tube, 6 mL Ficoll–Hypaque was added. Gently, the diluted blood was overlaid onto the Ficoll taking care not to mix the layers. The sample was then centrifuged at 500 *g* for 40 min at room temperature with the brake off to generate distinct plasma, PBMC, Ficoll, and red blood cell fractions. A pipette was inserted directly through the plasma layer to carefully harvest the PBMC layer via gentle aspiration and transfer it to a fresh 50 mL tube. The fraction was then washed using PBS and centrifuged at 500 *g* for 20 min at room temperature with the rotor brake on. The supernatant was then carefully decanted to leave a PBMC pellet at the base of the tube. The pellet was washed multiple times with PBS and pelleted using centrifugation for 400 *g* for 20 min. RNA was extracted and quantitative polymerase chain reaction (qPCR) was performed.

#### Transforming growth factor-β phospho antibody array

A Transforming Growth Factor (TGF)-β Signaling Phospho Antibody Array (Full Moon BioSystem PTG176) was used for specific phosphorylation profiling and screening with 176 antibodies linked to the TGF-β signalling pathway, as per the manufacturer’s protocol. Macrophages were exposed to PBS or to yREX3 for 48 h, and the absorbance A280 was determined using a UV spectrometer (e.g. Nanodrop); 80 OD (absorbance value reflecting the protein concentration of the lysate and a means of standardization for different samples across the phospho array), as measured by UV absorbance spectroscopy (A280) of cells, was used for each array by following the manufacturer’s indications.

#### Immunohistochemistry

Tissues were embedded in optimal cutting temperature (OCT) compound and frozen in 2-methyl butane pre-cooled in liquid nitrogen and then stored at −80°C until sectioning. Serial sections of the heart were cut at the mid-papillary level in the transverse plane. All sections were cut to between 5 and 6 μm using a cryostat (CM3050S, Leica) and adhered to superfrost microscope slides. Cryosections of the heart were fixed with 4% paraformaldehyde solution (Fisher Scientific, AAJ19943K2) for 10 min, washed with PBS, permeabilized with 0.2% Triton™ X-100 (Millipore Sigma, T8787), and blocked [Protein Block, Dako with 0.05% Saponin (Sigma-Aldrich, S4521)] for 30 min at room temperature. Following the 30 min block, the slides were then incubated overnight with primary antibodies diluted in blocking solution at 4°C. The primary antibodies are as follows: CD68 (1:100, Abcam ab125212 and Abcam ab955), Phospho-Smad3 (Ser213) (1:100, Invitrogen, # PA5-104942), alpha-sarcomeric actin (1:100, Abcam ab68167), Vimentin (1:100, Abcam ab24525). After the overnight incubation, the slides were washed with PBS (3 times 5 min) and incubated with the appropriate Alexa Fluor–conjugated secondary antibody (1:200, Invitrogen) for 2 h at room temperature. Following the secondary incubation, the slides were washed with PBS (3 times 10 min) and coverslips were mounted with Fluoroshield with DAPI (Sigma-Aldrich) mounting medium. The slides were imaged using fluorescence microscopy (Cytation 5, BioTek) and quantified using ImageJ software.

#### TUNEL assay

Tissues were embedded in OCT compound and frozen in 2-methyl butane pre-cooled in liquid nitrogen and then stored at −80°C until sectioning. Serial sections of the heart were cut at the mid-papillary level in the transverse plane. All sections were cut to between 5 and 6 μm using a cryostat (CM3050S, Leica) and adhered to superfrost microscope slides. Cryosections of the heart were fixed with 4% paraformaldehyde solution (Fisher Scientific, AAJ19943K2) for 10 min, washed with PBS, permeabilized for 30 min with 0.2% Triton™ X-100 (Millipore Sigma, T8787) and 0.5% bovine serum albumin, and rinsed two times in PBS for 5 min each. For the TUNEL reaction, samples were incubated with 100 µL (or enough to cover the sample) TUNEL equilibration buffer for 5 min. After removing the equilibration buffer, 50 µL (or enough to cover the sample) of TUNEL reaction mix was added to each sample, and tissue was incubated for 2 h at 37°C, protected from light. The samples were then rinsed three times in PBS-TB for 5 min each and co-labelled overnight at 4°C with alpha-sarcomeric actin (1:100, Abcam ab68167).

#### *In vitro* efferocytosis assay

Heart-derived H9C2 cells were labelled with DiO (Invitrogen) for 30 min at 37°C in the dark, washed, and then starved for 48 h using serum-free media. The resulting apoptotic cells were counted and added to the primary macrophage cultures. At the appropriate time point (20 or 60 min), the cells were washed two times with PBS, fixed, and then stained with Rhodamine Phalloidin (Invitrogen, R415) or CD68 (Abcam ab955) by following the manufacturer’s protocol, imaged by fluorescence microscopy (Cytation 5, BioTek), and quantified using ImageJ software.

### Statistical analysis

Statistical comparisons between any two groups were made using an independent one-tailed or two-tailed independent Student’s *t*-test with a 95% confidence interval. Comparisons made among three or more groups were made using a one-way analysis of variance with Tukey’s post-test to control for multiple comparisons or the Dunnett test. Statistical comparisons done on sequencing data were used for discovery purposes only. On their own, these results should be regarded as descriptive.

## Results

### yREX3 is enriched in human extracellular vesicles and is cardioprotective in myocardial infarction

**Figure 1:**
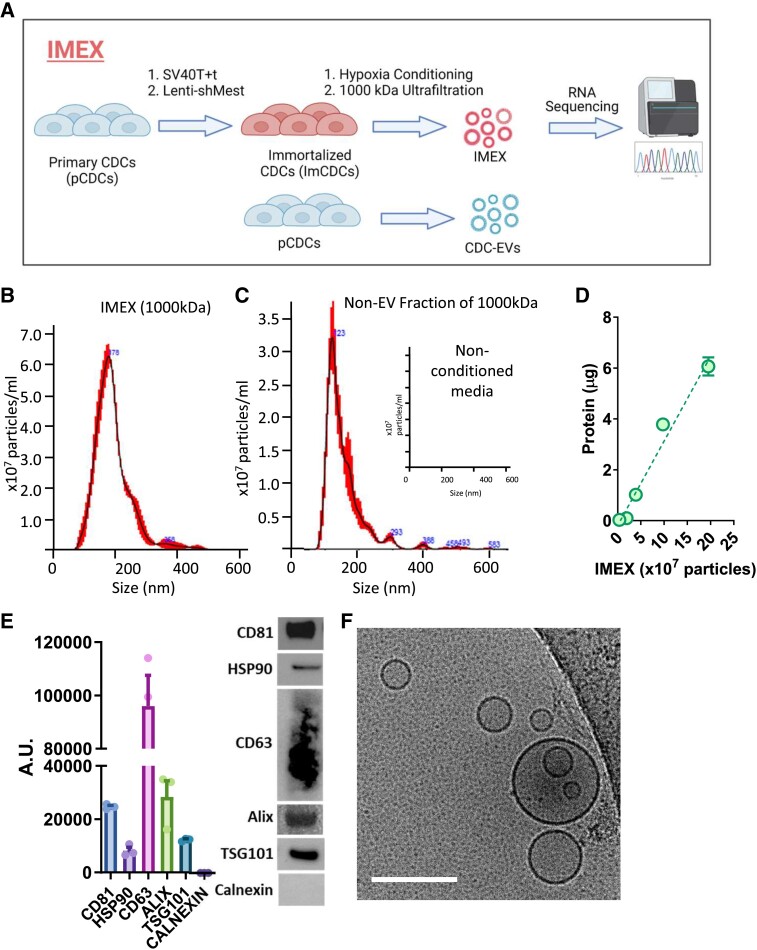
Characterization of extracellular vesicles from therapeutically enhanced cardiac stromal cells (IMEX). (*A*) Engineering of immortalized cardiosphere-derived cells (imCDCs) from primary cardiosphere-derived cells and preparation of extracellular vesicles from both (cardiosphere-derived cell extracellular vesicles and IMEX, respectively) for RNA sequencing. (*B*) Nanosight tracking analysis of particle size distribution and concentration of IMEX (1000 kDa), (*C*) non-extracellular vesicle fraction of 1000 kDa and non-conditioned culture media (used to produce extracellular vesicles: inset). (*D*) Extracellular vesicle purity is shown as particles (IMEX) vs. micrograms of protein. (*E*) Western blot for typical exosome markers (CD81, HSP90, CD63, Alix, TSG101). IMEX is negative for Calnexin (*n* = 3 samples/group, mean ± standard error of the mean). (*F*) IMEX visualized by cryotransmission electron microscopy. Scale bar: 200 nm

Immortalized human CDCs secrete cardioprotective EVs (IMEX,^14^*Figure 1A*) that, when isolated using ultrafiltration, are ∼100–300 nm in diameter by light scattering [nanosight tracking analysis (NTA): *Figure 1B*]. For comparison, the flow-through also included particles (like extruded EVs and protein complexes; *Figure 1C*), but no particles were present in non-conditioned media (*Figure 1C*, inset). Concentrated EVs (*Figure 1D*) had a marker profile (*Figure 1E*)^20^ and morphology by electron microscopy (*Figure 1F*) typical of the EV class known as exosomes.^21^ RNA sequencing of an equivalent particle number (as measured by NTA) of imCDCs and primary CDCs (less cardioprotective by comparison^14^) from the same donor identified generally comparable RNA distributions, but the cargo of their secreted EVs differed significantly: CDC-EVs had a higher proportion of tRNAs and micro RNAs (miRs), while IMEX were enriched in ribosomal RNAs and hairpin RNAs (see Supplementary data online, *Figure S1A* and *B*). The observed discrepancies in the distribution of different RNA classes between IMEX and primary CDC-EVs likely reflect the activation of beta-catenin in the parent cells of the IMEX, a genetic engineering step that enhanced therapeutic potency.^14^

**Figure 2:**
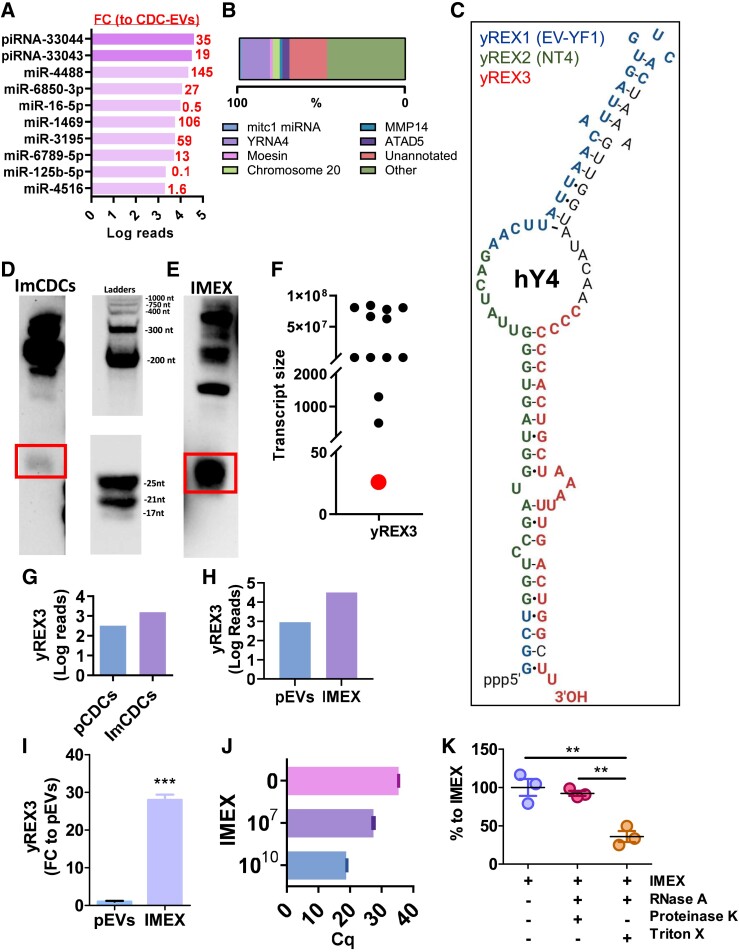
yREX3 is a small RNA enriched in IMEX. (*A*) Top 10 most expressed small non-coding RNAs in IMEX (expressed as log_10_ number of reads and fold change compared with primary cardiosphere-derived cell extracellular vesicles). (*B*) Per cent genomic origins of yREX3 show most sequence hits derive from the human YRNA 4 and its pseudogenes (lavender bar). (*C*) Alignment of yREX3 (in red) with the human YRNA 4 gene. Previously characterized small Y RNAs EV-YF1 and NT4 (renamed here as yREX1 and yREX2) are indicated in blue and green, respectively. (*D*) Northern blot analysis of yREX3 RNA expressed by immortalized cardiosphere-derived cells and (*E*) IMEX. (*F*) The sizes of individual RNA species are indicated. The band corresponding to the 26-nucleotide transcript is indicated in red. (*G*, *H*) Enrichment of yREX3 (in sequencing reads) in immortalized cardiosphere-derived cells to primary cardiosphere-derived cells. This enrichment was more pronounced in IMEX compared with primary cardiosphere-derived cell extracellular vesicles. (*I*) Quantitative polymerase chain reaction demonstrating the abundance of yREX3 in IMEX compared with primary cardiosphere-derived cell extracellular vesicles (*n* = 3 replicates per group, mean ± standard error of the mean, significance was determined using Student’s independent *t*-test, ***P < .001). (*J*) The abundance of yREX3 in IMEX was further confirmed in quantitative polymerase chain reaction by using 1 × 10^10^ and 1 × 10^7^ IMEX as input; data are expressed as Cq cycles in quantitative polymerase chain reaction and no IMEX (phosphate-buffered saline only, indicated as 0) used as a negative control. (*K*) yREX3 RNA is contained inside IMEX as shown by protection from RNase A degradation and proteinase K treatment (*n* = 3 biological replicates per group, mean ± standard error of the mean, significance was determined using one-way analysis of variance with Tukey’s post-test, **P < .01)

Sequencing of IMEX RNA further identified a plentiful 26-nucleotide RNA sequence indexed as piRNA-33044 and a second, similar sequence indexed as piRNA-33043 (*Figure 2A*). Neither was previously characterized. We focused on piRNA-33044 because of its high absolute read abundance as well as its unique enrichment in IMEX compared with CDC-EVs. Recognizing that most putative piRNA sequences in non-gonadal cells are misannotated, being piwi-independent fragments of other ncRNAs,^22^ we performed blast analysis to identify other possible genomic origins. The sequence indexed as piRNA-33044 overlapped significantly (92% homology) with the 3′ region of human Y RNA4 and its pseudogenes (*Figure 2B*). We refer to this small RNA as yREX3: a small Y RNA species (***yR***EX3) in exosomes (yR***EX***3) and the third member of its class to be characterized (yREX***3***). Following this new nomenclature convention, the previously identified Y RNA4 derivatives EV-YF1 and NT4 are renamed as yREX1 and yREX2, respectively (*Figure 2C*). Northern blot analysis of RNA isolated from imCDCs and IMEX confirmed the 26-nucleotide size of the small RNA, along with larger transcripts (*Figure 2D–F* and Supplementary data online, *Figure S1C*). yREX3 was enriched in both imCDCs and IMEX relative to CDCs and CDC-EVs, respectively, as shown by sequencing (*Figure 2G* and *H*) and confirmed by qPCR (*Figure 2I* and *J*). We also confirmed that yREX3 is contained within EVs using an RNAse and proteinase protection assay (*Figure 2K*). Since some Y RNAs can be associated with ribonuclear complexes,^9^ we included proteinase K to break down any proteins potentially associated with the RNA that may otherwise protect it from RNAse degradation.

**Figure 3:**
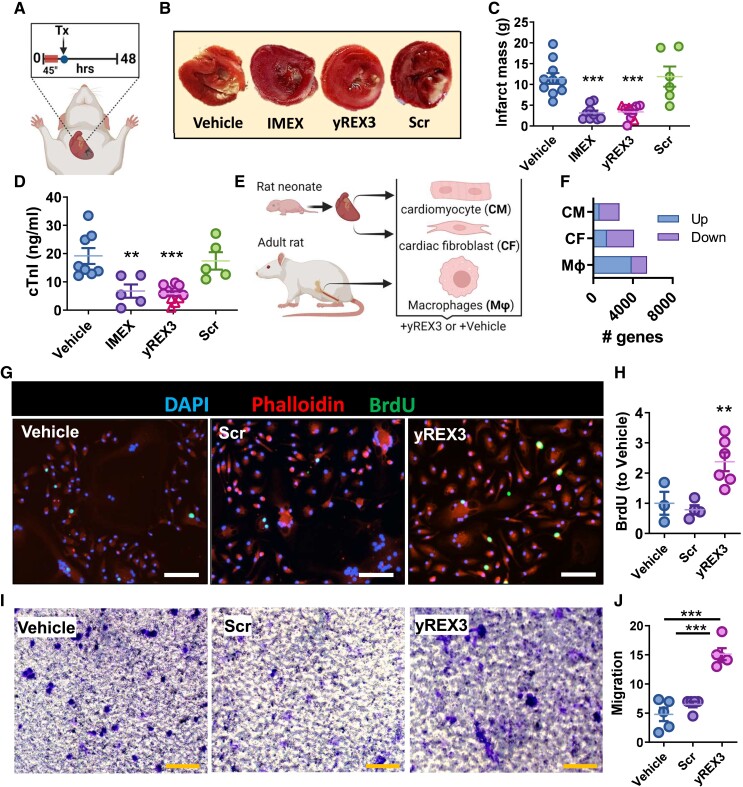
yREX3 is cardioprotective *in vivo* and enhances macrophage activation. (*A*) Rats underwent myocardial infarction (induced by 45 min of ischaemia followed by reperfusion) and 20 min after reperfusion they received saline, yREX3, IMEX, or scrambled sequence (Scr) intramyocardially. Forty-eight hours post-myocardial infarction, yREX3 showed cardioprotective activity as shown by reduced scar mass measured by 2,3,5-triphenyl-2H-tetrazolium chloride staining (representative images; *B*) and infarct mass quantification (*C*) and lower circulating cardiac Troponin I levels (*D*) compared with animals injected with vehicle or scramble (*n* = 6–10 animals/group, mean ± standard error of the mean). Alternatively, rats received a retro-orbital injection of yREX3 (400 ng/animal); pink triangles in *C* and *D* (all data presented as mean ± standard error of the mean, comparison between groups were evaluated using one-way analysis of variance with Tukey’s post-test with **P < .01 and ***P < .001). (*E*) Schematic of cell isolation from rat pups (cardiomyocytes and cardiac fibroblast) and the mother rat (bone marrow-derived macrophages (Mϕ). (*F*) Transcriptomic data of cardiomyocytes, cardiac fibroblasts, and BMDM (Mϕ) exposed to yREX3 (80 nM for 24 h) show significant differential gene expression compared with vehicle-exposed cells (*n* = 3 samples/group), expressed as the number of genes up- and down-regulated in the three cell types after *in vitro* exposure to yREX3. (*G*, *H*) *In vitro*, yREX3 enhances macrophage proliferation as analysed by BrdU incorporation (*n* = 3–6 replicates from two different experiments) and representative images (scale bar: 200 µm). (*I*, *J*) yREX3 potentiates macrophage migration at 24 h post-exposure using a Bowden chamber assay (migration calculated as integrated density/area, *n* = 5 independent experiments) and representative images (scale bar: 100 µm). All data presented as mean ± standard error of the mean. Comparison between groups was evaluated using one-way analysis of variance with Tukey’s post-test with **P < .01 and ***P < .001

To screen for disease-modifying bioactivity, we administered yREX3 encapsulated in LNPs, (400 ng/animal), IMEX (as a positive control, 1 × 10^10^ particles/animal), scramble LNPs (Scr, with the same nucleotide content as yREX3 but in random order verified to lack homology to rat or human genomes; 400 ng/animal), or vehicle (empty LNPs; Veh) 20 min post-reperfusion in rats with MI. These doses reflect previously identified effective doses of EVs (particularly IMEX and synthetic small RNA^17^). Tissues were harvested 48 h later for histology and circulating levels of cardiac Troponin I (cTnI; *Figure 3A*). Rats that had received yREX3 (or IMEX) showed much smaller infarcts (*Figure 3B* and *C*) and lower levels of cTnI compared with Veh or Scr (*Figure 3D*). Therapeutic responses were similar whether yREX3 was administered via the intracoronary (purple circles) or the intravenous route (pink triangles; *Figure 3C* and *D*), consistent with the notion that yREX3 exerts its effects systemically.

### yREX3 modulates the transcriptome of macrophages and enhances their proliferation and migration

To identify relevant target cells, we studied the transcriptomes of macrophages, cardiac fibroblasts, and cardiomyocytes in response to yREX3 *in vitro* (*Figure 3E*). Infiltrating macrophages, the primary target cells of EV therapy *in vivo*,^18,23^ are crucial to cardiac repair, while fibroblasts mediate scar formation and cardiomyocytes power the heartbeat. Unbiased analysis revealed varying yREX3-induced changes to the transcriptomes of all those cell types, with the most profound effects seen in macrophages (5435 genes vs. 4114 and 2653, respectively; *Figure 3F*). Relevant pathways affected include endothelial nitric oxide synthase (eNOS) signalling (see Supplementary data online, *Figure S2A–C*), G-protein-coupled receptor signalling (see Supplementary data online, *Figure S2D* and *E*), and interferon signalling (see Supplementary data online, *Figure S2F* and *G*). Consistent with previous observations, several markers of efferocytosis were up-regulated in yREX3-exposed macrophages (see Supplementary data online, *Figure S2H*). Given the particularly extensive transcriptomic effects of yREX3 on macrophages (see Supplementary data online, *Figure S2F* and *G*) and the pivotal role of macrophages in EV-mediated cardioprotection,^23–25^ we focused on this cell type. To verify that yREX3 was taken up by monocyte-derived macrophages *in vivo*, we quantified yREX3 levels in PBMCs in rats given yREX3 (intravenously) 15 min post-infusion. By qPCR, PBMCs isolated from rats that received yREX3 had a >5000-fold abundance compared with vehicle controls (see Supplementary data online, *Figure S2I*). yREX3 alters macrophage phenotype, enhancing their proliferation (as shown by BrdU staining; *Figure 3G* and *H*) and migration (by Boyden chamber assay; *Figure 3I* and *J*).

### yREX3 silences Pick1 expression through DNA methylation in macrophages

**Figure 4:**
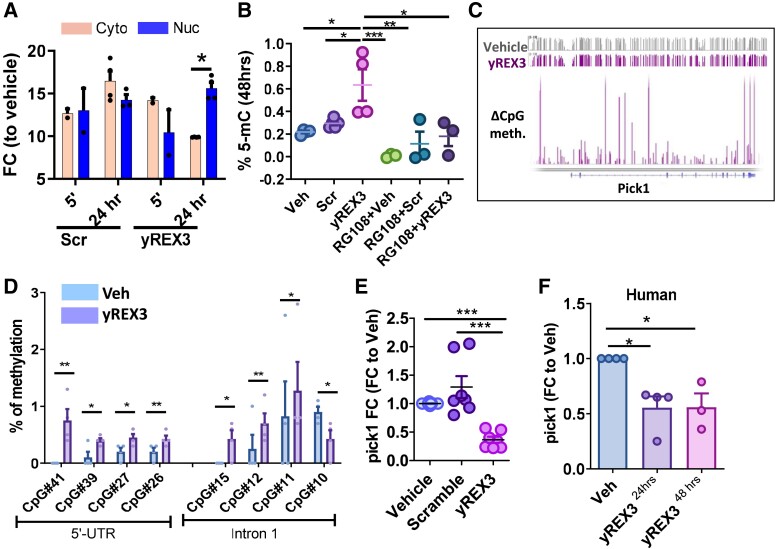
yREX3 methylates pick1 to enhance phagocytosis in macrophages. (*A*) yREX3 can shuttle between cytoplasm and nucleus in BMDM as shown by the quantitative polymerase chain reaction for yREX3 of cytoplasmic and nuclear RNA in BMDM transfected with yREX3 at different time points (data expressed as log_2_-fold change using snoU6 as housekeeping (scramble vs. vehicle and yREX3 vs. vehicle). (*B*) Analysis of global methylation levels in BMDM 48 h post-exposure to vehicle, yREX3, or scramble (Scr) with or without the DNA methyltransferase inhibitor RG108. (*C*) Methylation peaks for the Pick1 gene in yREX3-transfected BMDM (data presented as the difference in CpG-methylated regions vs. vehicle-exposed cells at 24 h). (*D*) Percentage of methylation in vehicle- and yREX3-exposed BMDM at 48 h in the different CpG sites was analysed (only relevant regions with different methylation profiles shown; *n* = 3–4 biological replicates/group). (*E*) Quantitative polymerase chain reaction for Pick1 expression levels in vehicle-, yREX3-, and scramble-exposed BMDM at 48 h (data presented as fold change compared with vehicle). (*F*) Quantitative polymerase chain reaction for Pick1 expression levels in vehicle- and yREX3-exposed human peripheral blood mononuclear cell–derived macrophages at 24 and 48 h (data presented as fold change compared with vehicle) (*A*–*C*, *E*, *F*). All data presented as mean ± standard error of the mean, comparison between groups were evaluated using a Student’s independent *t*-test and one-way analysis of variance with Tukey’s post-test with *P < .05, **P < .01, and ***P < .001. FC, fold change.

To probe the mechanism of yREX3’s effects on macrophage gene expression, we first quantified its subcellular localization. yREX3 was abundant in the cytoplasm soon after transfection into macrophages, but, by 24 h, had translocated to the nucleus, while Scr remained largely cytoplasmic (*Figure 4A*). Western blots of nuclear and cytoplasmic markers confirmed the purity of fractional preparations (see Supplementary data online, *Figure S3A*). Given its translocation to the nucleus, we hypothesized that yREX3 might exert epigenetic effects. Global methylation analysis showed that yREX3 selectively increased methylation of DNA relative to Veh or Scr (*Figure 4B*), an effect evident as early as 24 h (see Supplementary data online, *Figure S3B*) and waned by 72 h (see Supplementary data online, *Figure S3C*). The DNA methyltransferase (DNMT) inhibitor RG108 abrogated yREX3-mediated hypermethylation (*Figure 4B*). Whole-genome bisulphite sequencing revealed four differentially methylated genes (see Supplementary data online, *Figure S3D*). Among them, protein interacting with C kinase-1 (Pick1; hypermethylated; Supplementary data online, *Figure S3D*) caught our attention as an adaptor protein that binds to protein kinase C and several key membrane receptors and transporters. The fact that Pick1 influences interferon,^26^ GPCR,^27^ and eNOS^28^ signalling would rationalize the observed transcriptomic changes (see Supplementary data online, *Figure S2B–G*). Primer-specific methylation analysis pinpointed CpG islands in the 5′ untranslated region (UTR) and Intron 1 of the Pick1 locus (*Figure 4C* and *D* and Supplementary data online, *Figure S3E*). Consistent with the observed DNA methylation, yREX3 led to transcriptional repression of Pick1 (compared with Veh or Scr) in rat bone marrow–derived macrophages (*Figure 4E*) and human peripheral blood–derived macrophages (*Figure 4F*). Interestingly, yREX3 did not affect Pick1 expression in cardiomyocytes or cardiac fibroblasts (murine and human), which points to a cell-type-specific mechanism (see Supplementary data online, *Figure S3F–H*). Therefore, yREX3 regulates Pick1 in macrophages through DNA methylation–mediated gene silencing. In macrophages where Pick1 alone was silenced, global methylation levels were also increased, although not to the same extent as after yREX3 exposure (see Supplementary data online, *Figure S3I*). These findings suggest that global methylation is perhaps partially driven by indirect or downstream effects of yREX3 signalling. The DNMT3a and DNMT3b methyltransferases and the DNMT3-like were found enriched in RNA-protein pull-down samples in yREX3-exposed macrophages when compared with scramble (see Supplementary data online, *Figure S3J*).

### Pick1 silencing by yREX3 potentiates Smad3 signalling and enhances efferocytosis

**Figure 5:**
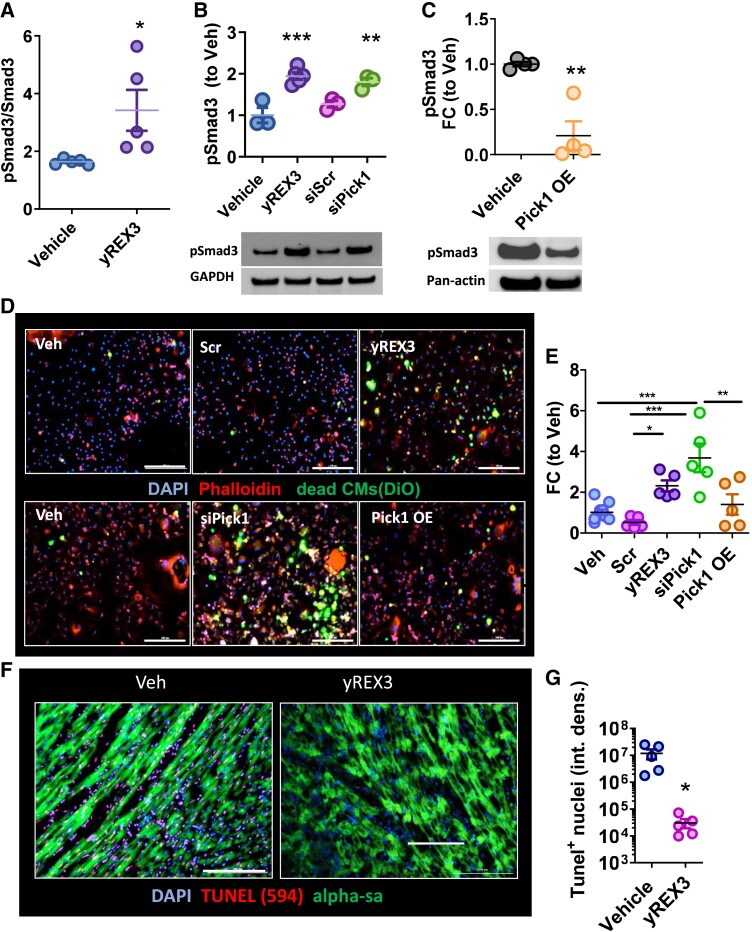
Pick1 suppression activates Smad3 and enhances efferocytosis in macrophages. (*A*) Phosphorylation levels of Smad3 expressed as phosphorylated/total in vehicle- and yREX3-exposed BMDM (*n* = 5–6 technical replicates/group). (*B*) Western blot showing increased phosphorylation of Smad3 (Ser213) in macrophages after exposure to yREX3 or a small interfering RNA targeted against Pick1 (siPick1) compared with scramble (siScr) or vehicle (*n* = 3–5 biological replicates per group). (*C*) Western blot showing decreased phosphorylation of Smad3 (Ser213) in macrophages after exposure to a Pick1 overexpressing vector (Pick1 OE) at 24 h compared to vehicle (*n* = 4 biological replicates per group). (*D* and *E*) Efferocytosis assay showing increased uptake of DiO-labelled dead rat cardiomyocytes by yREX3-exposed or small interfering RNA targeted against Pick1–exposed macrophages compared to those exposed to vehicle, Scr, or Pick1 overepxression (representative images taken at 48 h, *n* = 3–6 biological replicates/group). (*F* and *G*) TUNEL assay in rat heart sections 48 h after myocardial infarction in animals receiving vehicle or yREX3 i.v. administration and representative images. All data presented as mean ± standard error of the mean, comparison between groups using Student’s independent *t*-test or one-way analysis of variance with Tukey’s post-test with *P < .05, **P < .01, and ***P < .001

Pick1 activates macrophage polarization by regulating Smad signalling.^29^ Protein array analysis of macrophage lysates showed that yREX3 exposure increased Smad3 phosphorylation at Serine-213 (Ser213; *Figure 5A*), and reduced Smad1 (see Supplementary data online, *Figure S4A*) and Smad2 (see Supplementary data online, *Figure S4B*) phosphorylation, compared with Veh. Indeed, yREX3- or small interfering RNA (siRNA)-mediated silencing of Pick1 led to Smad3 hyperphosphorylation compared with respective controls (*Figure 5B*). Although Smad3 is part of the TGF-β signalling pathway, yREX3 exposure in macrophages led to a modest increase in TGF-β gene expression and no change in protein secretion (see Supplementary data online, *Figure S4C* and *D*). These findings confirm that yREX3-mediated Smad3 activation is independent of TGF-β secretion. We also verified the expression levels of PKC isoforms α and ε, but we did not find any significant increase in yREX3-exposed macrophages (see Supplementary data online, *Figure S4E*). As a consistency check, Pick1 depletion (see Supplementary data online, *Figure S5A*) triggered macrophage proliferation comparably with yREX3 exposure (see Supplementary data online, *Figure S5B*). Pick1 overexpression (see Supplementary data online, *Figure S5C–E*) did not induce macrophage proliferation (see Supplementary data online, *Figure S5F*). Overexpression of Pick1 also decreased Smad3 phosphorylation (*Figure 5C*). Smad3 activation in macrophages enhances clean-up phagocytosis (i.e. efferocytosis),^30^ so we were not surprised to find that yREX3 enhanced efferocytosis of dead cardiac cells *in vitro*, an effect mimicked by Pick1 depletion (*Figure 5D* and *E*). The increase in efferocytosis was reversed when Pick1 was overexpressed (*Figure 5D* and *E*). Human macrophages exposed to yREX3 also show an increase in their migratory capacity and an increase in efferocytosis (see Supplementary data online, *Figure S5F* and *G*).

Given the prominence of macrophages in EV-mediated cardiac repair,^15,16^ we probed the role of macrophages in yREX3-induced cardioprotection. yREX3 exposure *in vivo* did not lead to a significant increase in macrophage proliferation in infarcted heart tissue (see Supplementary data online, *Figure S6A* and *B*. cf. *in vitro*, *Figure 3G* and *H*), consistent with the inflammatory state of post-ischaemic cardiac tissue and its already-enhanced macrophage proliferation. Nevertheless, Smad3 phosphorylation was increased in yREX3-exposed hearts (see Supplementary data online, *Figure S6A* and *C*), consistent with the macrophage-specific effect of yREX3 on Smad3 phosphorylation (*Figure 5A* and *B*). Smad3 phosphorylation seems to be specifically associated with CD68-positive cells in yREX3-treated animals, while in vehicle-treated animals, it associates also with vimentin-positive cells; cardiomyocytes do not express pSmad3 in any of the treatment conditions (see Supplementary data online, *Figure S6D*). The increase in efferocytosis was recapitulated in yREX3-exposed post-MI hearts, as demonstrated by a decrease in TUNEL-positive nuclei in the infarct zone, 48 h after MI (*Figure 5F* and *G*). Taken together, these findings support the concept that yREX3 enhances macrophage efferocytosis through Pick1 suppression and downstream activation of Smad3.

### yREX3 complexes with PTBP3, RAVER1, and DNA methyltransferases to epigenetically silence Pick1

**Figure 6:**
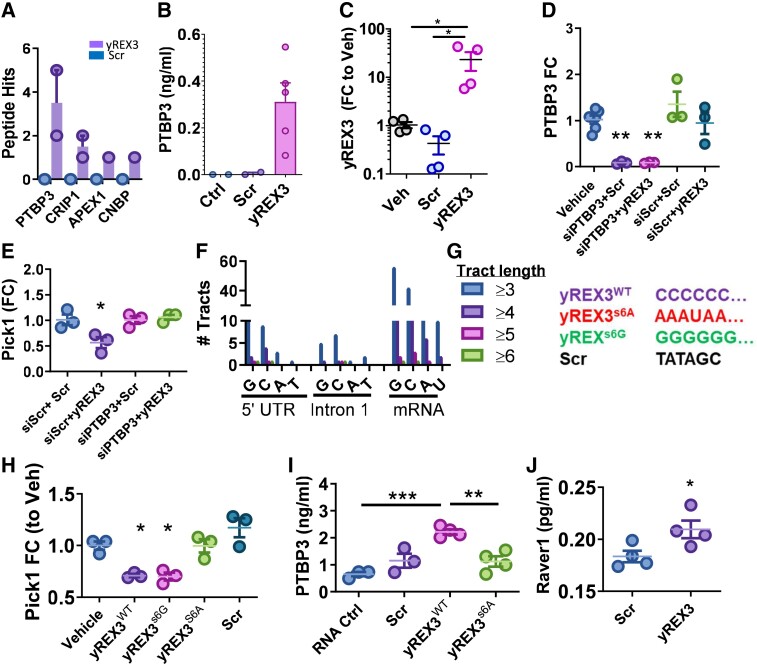
yREX3 binds PTBP3 through a six-cytosine motif. (*A*) Mass-spectrometry analysis of RNA-protein pull-down showing peptide hits identified in yREX3- and scrambled-exposed macrophages. (*B*) Enzyme-linked immunosorbent assay for PTBP3 in RNA-protein pull-down experiments using control (vehicle) or scrambled sequence and yREX3. (*C*) Quantitative polymerase chain reaction demonstrating yREX3 expression when PTBP3 is immunoprecipitated in macrophages exposed to vehicle, scramble, or yREX3 *in vitro*. (*D*) Quantitative polymerase chain reaction demonstrating down-regulation of PTBP3 after transfection of macrophages with a small interfering RNA targeted against PTBP3 (50 nM) for 48 h. (*E*) Quantitative polymerase chain reaction showing that polypyrimidine tract in yREX3 is essential for suppression of Pick1. (*F*) Analysis of the abundance of single-nucleotide tracts in the 5′ untranslated region, Intron 1 (where CpG sites are methylated), and in mRNA for Pick1 showing they are enriched in cytosine and guanine tracts compared with adenine and thymine (or uracil in the case of mRNA). (*G*) First six residues at the 5′ end in the yREX3 sequence (here identified as yREX3^WT^) are cytosines. Oligonucleotides with mutations in the first six residues at the 5′ end and for the scramble sequence. (*H*) Quantitative polymerase chain reaction for Pick1 expression levels in vehicle, yREX3, yREX3 with a silent mutation (yREX3s6A), yREX3 with a dysfunctional mutation (yREX3s6G), and scramble-exposed BMDM at 48 h (data presented as fold change compared with vehicle). (*I*) Enzyme-linked immunosorbent assay of PTBP3 in RNA-Protein pull-down experiments using control or scramble, yREX3, and yREX3 with the dysfunctional mutation (yREX3s6G). (*J*) Enzyme-linked immunosorbent assay of RAVER1 in immunoprecipitation experiments using scramble or yREX3 macrophage lysates (*A*–*D*, *G*–*I*). Pooled data presented as mean ± standard error of the mean, comparison between groups using one-way analysis of variance with Tukey’s post-test with *P < .05, **P < .01, and ***P < .001, or Student’s independent *t*-test (*H*)

Little is known about small Y RNA signalling. RNA-protein pull-down using biotinylated yREX3 followed by mass spectrometry of co-precipitated proteins identified polypyrimidine tract binding protein 3 (PTBP3/ROD1; *Figure 6A*), as further validated by ELISA (*Figure 6B*). Conversely, qPCR of immunoprecipitated PTBP3 demonstrated an abundance of yREX3 (*Figure 6C*). The association of Y RNA4 to PTBP proteins has been described, but this is the first demonstration that this region, in exclusion of the full-length Y RNA4, binds PTBP.^31^

Polypyrimidine tract binding protein 3 is involved in post-transcriptional processing of RNA via association with its ligand, RAVER1.^32^ Knock-down of PTBP3 (*Figure 6D*) resulted in loss of yREX3-mediated Pick1 suppression, demonstrating a critical upstream role of PTBP3 in yREX3 function (*Figure 6E*). Both cardiac fibroblasts and cardiomyocytes express lower levels of PTBP3 than seen in macrophages (see Supplementary data online, *Figure S7A*), comparable levels of RAVER1 (see Supplementary data online, *Figure S7B*), and higher expression of its suppressor RAVER2 (see Supplementary data online, *Figure S7C*), all of which rationalizes the lack of yREX3-dependent Pick1 suppression in those two cell types compared with macrophages (cf. *Figure 4E* and Supplementary data online, *Figure S3F–H*). Since PTBP3 binds to conserved stretches of pyrimidine-rich intronic sequences in pre-mRNA, we surmised it may bind yREX3 at cytosine repeats in the pre-mRNA of Pick1. The 5′ UTR, Intron 1 (where CpG sites are methylated), and the mRNA for Pick1 were all enriched in cytosines and guanines (*Figure 6F*), suggesting a putative recognition site via yREX3’s six-cytosine stretch at the 5′ end (yREX3^WT^; *Figure 6G*). To probe yREX3 binding, we generated two mutants wherein the six-cytosine leader sequence was substituted with purine stretches of either guanine (yREX3^s6G^) or adenine (yREX3^s6A^; *Figure 6G*). Surprisingly, yREX3^s6G^ showed preserved function, as demonstrated by Pick1 suppression capacity comparable with that of yREX3^WT^. However, yREX3^s6A^ showed loss of function (*Figure 6H*), and pull-down analysis confirmed loss of binding to PTBP3 (*Figure 6I*). We also confirmed RAVER1 binding after PTBP3 immunoprecipitation in macrophages exposed to yREX3 (*Figure 6J*). Thus, via its six-cytosine leader sequence, yREX3 binds the PTBP3/RAVER1 complex, which in turn suppresses Pick1.

### Adoptive transfer of macrophages exposed to yREX3 or protein interacting with C kinase-1-small interfering RNA is cardioprotective in myocardial infarction

**Figure 7:**
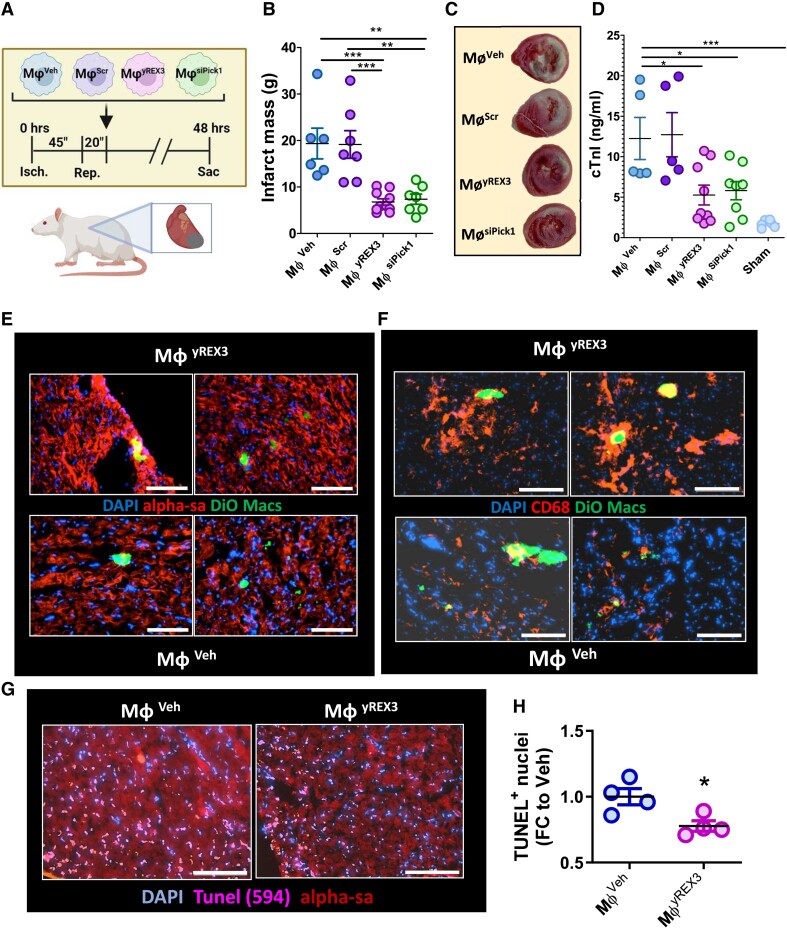
Macrophages mediate the cardioprotective effects of yREX3. (*A*) Schematic of the experiment design: macrophages exposed for 24 h to yREX3 (Mϕ^yREX3^; 80 nM), scramble (Mϕ^Scr^; 80 nM), vehicle (Mϕ^Veh^) or small interfering RNA targeted against Pick1 (Mϕ^siPick1^; 50 nM) were injected via the tail vein of the animals with myocardial infarction, 20 min after reperfusion. (*B*) At 48 h, rats infused with Mϕ^yREX3^ and Mϕ^siPick1^ showed cardioprotection as shown by reduced scar size (2,3,5-triphenyl-2H-tetrazolium chloride (TTC; *B, C*) and lower cardiac troponin levels at 48 h after *I*/*R* (*D*; *n* = 5–9 animals/group) compared to rats infused with Mϕ^Scr^ or Mϕ^Veh^. (*E*) In another set of experiments, BMDM-derived macrophages exposed to yREX3 and vehicle were stained for 30 min with DiO prior to tail vein injections in a rat model of *I*/*R*, and rats sacrificed after 4 h. Immunofluorescence of heart sections show the localization of DiO-positive cells into the infarct area of the animals. (*F*) Immunostaining of heart sections show DiO-positive cells are double positive for CD68 (marker of macrophages) and they localize together with other macrophages into the infarct area. (*G*, *H*) TUNEL assay in rat heart sections 4 h after myocardial infarction in animals receiving macrophages exposed to vehicle or yREX3 and representative images. (*B*, *D*) All data are presented as mean ± standard error of the mean, and comparison between groups was evaluated using a one-way analysis of variance with (*B*) Tukey’s post-test with *P < .05, or (*D*) Dunnett’s post-test vs. Mϕ^Veh^ with *P < .05, **P < .01, and ***P < .001; scale bar: 100 μm

We next tested whether the adoptive transfer of yREX3-conditioned macrophages could mimic yREX3-induced cardioprotection. Macrophages conditioned *ex vivo* by exposure to yREX3 (Mϕ^yREX3^), vehicle (Mϕ^Veh^), or Scr (Mϕ^Scr^) were infused into rats post-MI (*Figure 7A*). At 48 h, animals that had received Mϕ^yREX3^ had reduced infarct mass and cTnI levels compared with Mϕ^veh^ or Mϕ^Scr^ (*Figure 7B–D*). The cardioprotective benefits were mimicked by infusion of macrophages conditioned by exposure to a siRNA against Pick1 (Mϕ^siPick1^; *Figure 7B–D*). Thus, adoptive transfer of yREX3-conditioned macrophages replicated the cardioprotective effects of yREX3 itself (cf. *Figure 3A–D*), an effect reproduced by infusion of macrophages in which Pick1 had been silenced selectively. To examine the homing of macrophages to the infarct, hearts from another set of experiments were imaged after 4 h. While both Mϕ^veh^ and Mϕ^yREX3^ were found within the infarct area (*Figure 7E* and *F*) only animals receiving Mϕ^yREX3^ exhibited reductions in TUNEL-positive nuclei (*Figure 7G* and *H*). Therefore, Pick1 suppression in macrophages underlies yREX3-mediated cardioprotection.

## Discussion

We have identified a small ncRNA, yREX3, as having major cardioprotective effects (reductions of infarct mass and circulating cTnI levels) when given post-MI in an ischaemia-reperfusion model. The discovery of yREX3 was the result of an unbiased investigation of small RNAs plentiful in cardioprotective human EVs. yREX3’s ability to dramatically reduce MI-induced cardiac damage *in vivo* motivated us to probe its mechanism of action. We found that yREX3, in association with PTBP3, RAVER1, and DNMT, induces methylation of upstream CpG sites in the Pick1 locus. Mutational analysis identified the 5′ cytosine repeat motif as crucial in binding PTBP3. This may explain why yREX3 shuttled into the nucleus, while the scramble sequestered it in the cytoplasm. We surmise that yREX3 likely binds cytosolic PTBP3, which can then translocate to the nucleus to exert its signalling,^33^ while the scramble, lacking a coherent PTBP3 binding motif, remains in the cytosol, but we have not tested this conjecture. Silencing Pick1 activates Smad3 to enhance macrophage efferocytosis. The central role of macrophages was driven home by the demonstration that the adoptive transfer of yREX3-conditioned macrophages recapitulated the cardioprotective effects of yREX3, as did the infusion of Pick1-deficient macrophages.

This novel mechanism (*Structured Graphical Abstract*) of an obscure class of small ncRNA opens up several questions that logically remain to be addressed. These include: what is the recognition motif along yREX3 that determines binding to the Pick1 locus? What other activities of yREX3 contribute to the global increase in methylation (which cannot be explained by Pick1 methylation alone)? More broadly, what other small ncRNAs might regulate DNA methylation through the PTBP3/RAVER1 complex? (In unpublished data, we have excluded similar signalling by EV-YF1 or NT4.) Finally, which RNA enzymes process and load the mature sequence into the target locus? Dicer1 and Dicer2 are critical for the processing of siRNAs and miRNAs into RNA-induced silencing complexes^34^ but Dicer was not detected in yREX3 pull-downs. Given that PTBP3 is an RNA-processing protein, it is logical to examine the role of PTBP3 and its complex partners in processing and loading small DNA-methylating RNAs like yREX3 to the methylation complex, but such experiments are beyond the scope of the present study. It is important to contextualize the results with the current injury model. yREX3 may have signalling or gene-targeting modalities beyond targeting Pick1. In other models of injury, yREX3 may signal through a different mediator, including other genes identified in the whole-genome bisulphite sequencing data (cf. Supplementary data online, *Figure S3D*). However, in the context of ischaemic injury, the bioactivity of yREX3 is driven in large part by Pick1 suppression as demonstrated by adoptive transfer studies in which knocking in Pick1 in yREX3-exposed macrophages curtails the cardioprotective effects of the macrophages (cf. *Figure 7B–D*).

An important additional caveat is the short-term nature of the post-MI outcomes in the present study. Long-term follow-up, as well as testing in more clinically realistic large-animal models, will be necessary to advance yREX3 towards the clinic.

Its DNA-methylating activity implicates yREX3 as the index case for a new class of ncRNA, with a mechanism of action differing fundamentally from that of siRNAs, small hairpin RNAs (shRNAs), antisense oligonucleotides (ASOs), aptamers, and miRs. Unlike siRNAs, shRNAs, and miRs, which function by targeting messenger RNAs, or aptamers that bind protein active sites, yREX3 functions by methylating gene targets. We propose the term *s*mall *m*eth*y*lating RNA (smyRNA) for this new class of ncRNA. Another added nuanced dimension to yREX3 is its selective action on macrophages; it does not suppress Pick1 in cardiomyocytes or cardiac fibroblasts (see Supplementary data online, *Figure S3F–H*). Such selectivity would not be observed with siRNAs, shRNAs, or ASOs. Selective gene suppression is key in the injured microenvironment, where phenotypic outcomes of gene augmentation or suppression are cell-type specific (cf. Supplementary data online, *Figure S3F–H*).

More broadly, the discovery arc for yREX3 further validates the concept^4^ of screening obscure but plentiful ncRNAs from EV cargo for disease-modifying bioactivity. Using this paradigm, we have identified not only three YRNA species (yREX1, yREX2, and yREX3) but also a transfer RNA fragment plentiful in CDC-EVs,^35^ and a long ncRNA,^36^ as novel therapeutic candidates.

## Conclusions

In this study, we describe the mechanism and cardioprotective role of a small Y RNA mined from the cargo of cardioprotective EVs. yREX3 functions through targeted epigenetic gene silencing in macrophages to enhance their tissue reparative capacity post-MI. The fact that yREX3 is dramatically cardioprotective (>70% reduction in infarct mass, *Figure 3C*), even when administered 20 min after reflow, makes it well-suited for clinical application: it need not be given until primary PCI has been successfully performed. Indeed, no intervention currently exists that has demonstrated a reduction in infarct size when given after ischaemic injury.^1^ Drugs such as nitrates, beta-blockers, and others that enhance reperfusion fail to affect scar size in the long term.^37^ In addition, no clinically approved therapeutic leads to the dramatic reduction in infarct size observed with yREX3. Infarct size is a robust determinant of downstream complications including arrhythmia and heart failure.^38^ Therefore, therapeutic candidates that shrink scar size after reperfusion represent a disruptive leap in cardioprotective therapy.^1^ If yREX3 turns out to be efficacious in follow-up studies quantifying long-term outcomes post-MI in relevant pre-clinical models, there may be new hope in the as-yet futile search for cardioprotective agents^1^ adjunctive to the only therapy so far demonstrated to decrease infarct size in humans: recanalization of the culprit vessel.

## Supplementary Material

ehae357_Supplementary_Data

## Declarations

### Data Availability

All data are available in the main text or the supplementary data.
