# Supplementary material for: Non-coding RNA yREX3 from human extracellular vesicles exerts macrophage-mediated cardioprotection via a novel gene-methylating mechanism
Source: Eur Heart J. 2024 Jun 12;45(29):2660–73. doi: 10.1093/eurheartj/ehae357 (PMC11297535; doi:10.1093/eurheartj/ehae357)
Supplement: ehae357_Supplementary_Data [file ehae357_supplementary_data.docx]

**Supplementary material online**

**Table of contents**

| Supplemental Methods | 1 |
| --- | --- |
| Supplementary Figure Legends | 11 |
| Supplementary Figures | 13 |
|  |  |
|  |  |

**Supplemental Methods**

**RNA purification and qPCR analysis.** Total RNA, including small RNAs of approximately 18 nucleotides and greater were purified using the miRNeasy Mini Kit (Qiagen) according to the manufacturer’s recommended protocol. Reverse transcription was performed using High-Capacity RNA to cDNA (Thermo Fisher Scientific) or Taqman® microRNA Reverse Transcription Kit (Applied Biosystems) per the manufacturer’s protocol. Real-time PCR was performed using Taqman Fast Advanced Master Mix and appropriate Taqman Gene expression Assay (Thermo Fisher Scientific). The reaction was performed in QuantStudioTM 12K Flex Real-Time PCR System, and each reaction was performed in triplicate samples and adjusted using hprt1 and snu6 for mRNA and piRs respectively. (Life technologies). Cycling conditions were performed according to the Tagman protocol. Where appropriate, the 2^–ΔΔCt^ method was used to determine gene expression fold change.

**Nuclear/Cytosolic RNA isolation and qPCR analysis.** Nuclear and cytosolic RNA was purified using the Cytoplasmic and Nuclear RNA Purification kit (Norgen Biotek) according to the manufacturer’s recommended protocol. Reverse transcription was performed using Taqman® microRNA Reverse Transcription Kit (Applied Biosystems) per the manufacturer’s protocol, using specific primers for yREX3 and scramble sequence. Real-time PCR was performed using Taqman Fast Advanced Master Mix and appropriate Taqman Gene expression Assay (Thermo Fisher Scientific). The reaction was performed in QuantStudioTM 12K Flex Real-Time PCR System, and each reaction was performed in triplicate samples and adjusted using snu6 as housekeeping. (Life Technologies). Cycling conditions were performed according to the Taqman protocol. Where appropriate, the 2^–ΔΔCt^ method was used to determine gene expression fold change.

**Small RNA Sequencing.** Cell and EV RNA samples were sequenced at the Cedars-Sinai Genomics Core. Total RNA and Small RNA were analyzed using an Illumina NextSeq 500 platform for cell and EV samples, respectively.

**Northern Blot.** Total RNA from cells, including small RNAs of approximately 18 nucleotides and greater were purified using the miRNeasy Mini Kit (Qiagen) according to the manufacturer’s recommended protocol. Total RNA from EVs was isolated using a urine EV RNA isolation kit (Norgen Biotek, cat# 47200) according to the manufacturer’s recommended protocol. RNA isolated from cells and EVs (5µg of total RNA was used for cells, 2ug for EVs) was loaded in 15% TBE-Urea Gels (Invitrogen, EC6885BOX) using Novex TBE-Urea Sample Buffer (Invitrogen, LC6876) in a ratio 1:1. Both Biotinylated sRNA Ladder (Kerafast) and microRNA Marker (New England BioLabs) were loaded. After the run, SYBR Green II RNA Gel Stain (Invitrogen, S7564) was used to stain the gel. The RNA was then transferred from the gel to a positively charged nylon membrane (BrightStar™–Plus Positively Charged Nylon Membrane, Invitrogen) using downward transfer for 30 minutes at 200mA. After cross-linking, the membrane was hybridized using a probe specific for yREX3 (15 ng/ml final concentration, Customed Qiagen miRCURY LNA miRNA Detection Probe 339111) overnight at 42° C and detected using Chemiluminescent Nucleic Acid Detection Module Kit (Thermo Fisher 89880). The bands were analyzed and quantified using ImageJ.

**Methylation Analysis**

**Global methylation ELISA.** Macrophages were exposed to PBS or yREX3 and scrambled-sequence for 24 hours, 48 hours, or 72 hours (48 hours transfection, followed by 24 hours recovery). Genomic DNA was isolated using the DNeasy Blood & Tissue Kit (Qiagen) per the manufacturer’s protocol. Global DNA methylation levels were assessed using MethylFlash Global DNA Methylation (5-mC) ELISA Easy Kit (Epigentek) using 100ng of input DNA for all conditions. Results are expressed as a percentage of methylated DNA (%5-mC). In another set of experiments, macrophages were exposed to PBS, yREX3, and siPick1 for 24 hours, genomic DNA was isolated and the global DNA methylation levels were analyzed using the same ELISA kit.

**Whole Genome Bisulfite Sequencing.** Genomic DNA was isolated using the DNeasy Blood & Tissue Kit (Qiagen) per the manufacturer’s protocol. Bisulfite sequencing was performed at the Cedars-Sinai Genomics Core. FASTQ files were analyzed.

**Pick1-targeted methylation.** NGS methylation assays were designed to interrogate the DNA methylation status of 56 CpG sites in the 5’ Upstream to Intron 6 regions of the Rat Pick1 gene in macrophages exposed to PBS or to yREX3 for 24 hours and 48 hours. The CpG loci location or coordinates are based on Ensemble Gene ID ENSRNOG00000011507, Ensemble transcript ID ENSRNOT00000016077, and Rnor_6.0 genomic build. Extracted genomic DNA with OD260/280 between 1.8 and 2.2 was used in the analysis. Bisulfite modification was carried out using EZ DNA Methylation Kit™ (Cat.#D5001) per the manufacturer’s protocol (Zymo Research, Irvine, CA) with minor modifications. 200-500 ng of gDNA were used for bisulfite modification. The bisulfite-modified DNA samples were eluted using M-elution buffer in 46 µL. All bisulfite-modified DNA samples were amplified using separate multiplex or simplex PCRs as listed in Section 2.1. PCRs included 0.5 units of HotStarTaq (Qiagen; Hilden, Germany; cat# 203205), 0.2 µM primers, and 3 µL of bisulfite-incubated DNA in a 20 µL reaction. PCR cycling conditions were the following: 95°C 15 min; 45 x (95°C 30s; Ta°C 30s; 68°C 30s); 68°C 5 min; 8°C ∞. All PCR products were verified using the Qiagen QIAxcel Advanced System (v1.0.6). Before library preparation, PCR products from the same sample were pooled and then purified using the QIAquick PCR Purification Kit columns or plates (Qiagen cat# 28106 or 28183). Libraries were prepared using a custom Library Preparation method created by EpigenDx. Next, library molecules were purified using Agencourt AMPure XP beads (Beckman Coulter; Brea, CA; cat# A63882). Barcoded samples were then pooled in an equimolar fashion before template preparation and enrichment on the Ion Chef™ system using Ion 520™ & Ion 530™ ExT Chef reagents (Thermo Fisher; Waltham, MA; cat# A30670). Following this, enriched, template-positive library molecules were sequenced on the Ion S5™ sequencer using an Ion 530™ sequencing chip (cat# A27764). FASTQ files from the Ion Torrent S5 server were aligned to the local reference database using open-source Bismark Bisulfite Read Mapper with the Bowtie2 alignment algorithm. Methylation levels were calculated in Bismark by dividing the number of methylated reads by the total number of reads. The analysis was performed by EpigenDx (Hopkinton, MA).

**Cell and EV lysates and protein assay.** Cell and EV lysates were collected for western blot. Cells were pelleted and resuspended in 1× RIPA buffer (Pierce) supplemented with a protease/phosphatase inhibitor cocktail on ice for 30 min. EVs resuspended in PBS were lysed in 10× RIPA buffer (Pierce) with protease inhibitor on ice for 30 min. Protein lysates were isolated by centrifugation at 14,000 rpm for 15 min at 4°C. Protein concentration was measured using a DC Protein Assay kit (Bio-Rad).

**Western blot.** Membrane transfer was performed using the Turbo Transfer System (BIO-RAD) after gel electrophoresis. The subsequent antibody staining was then applied and detected by Super Signal West Pico PLUS Chemiluminescent Substrate (Thermo Fisher Scientific). Antibodies used in this study are the following. For EVs markers: CD81 (Invitrogen MA5-13548), HSP90 (Abcam Ab13492), CD63 (Proteintech # 25682), Alix (Invitrogen MA1-83977), TSG101 (Invitrogen (MA1-23296), Calnexin (Cell Signaling C5ca), Anti-Rabbit IgG, HRP-Linked Antibody (Cell Signaling Technology #7074), Anti-Mouse IgG, HRP-Linked Antibody (Cell Signaling Technology #7076). For cells: Phospho-Smad3 (Ser213) (Invitrogen # PA5-104942), pick1 (Invitrogen™, PIPA576084), GAPDH Rabbit mAb, HRP Conjugate (Cell Signaling #3683), Pan-Actin (D18C11) Rabbit mAb, HRP Conjugate (Cell Signaling #12748), Anti-Rabbit IgG, HRP-Linked Antibody (Cell Signaling Technology #7074) For nuclear and cytosolic fractions: H3A (Cell Signaling #4499) and Pan-Actin (D18C11) Rabbit mAb, HRP Conjugate (Cell Signaling #12748), respectively were used. Immunoreactivity was visualized using a ChemiDoc (Bio-Rad). Protein abundance was analyzed using ImageJ software.

**Cell isolation**

**Rat bone marrow-derived macrophage (BMDM) isolation.** Femurs were isolated from 7‐ to 10‐week‐old Wistar–Kyoto rats. Bone marrow was isolated by flushing with PBS and then filtering through a 70‐μm mesh. Red blood cells were lysed with ACK buffer (Invitrogen) and then resuspended in IMDM (Gibco) containing 10% FBS, 0.5% gentamycin, and 20 ng/ml M‐CSF (eBioscience) for plating. The media was exchanged every 2–3 days until day 5, at which point bone marrow‐derived macrophages (BMDMs) were used for *in vitro* experiments.

**Neonatal rat ventricular myocyte isolation.** Neonatal rat cardiomyocytes were isolated from P2 neonatal Sprague–Dawley rats as described^8^. The cells were plated on fibronectin-coated 12-well plates at a density of 0.5 million cells/well in Dulbecco’s Modified Eagle Medium (DMEM) containing 10% Fetal Bovine Serum (Gibco) media, and incubated at 37°C, with 5% CO_2_ for 24 hrs.

**Rat cardiac fibroblast isolation.** Cardiac fibroblasts were isolated from the first and second pre-plating from NRVM isolation procedure and cultured in DMEM containing 10% Fetal Bovine Serum (Gibco), and incubated at 37°C, with 5% CO_2_ until confluence and used at p2 and p3.

**Human macrophages.** Human Peripheral Blood Monocytes (PBMCs, Stem cell technologies, # 70034) were plated in 12-well plates at a concentration of 2x10^6^ cells/ml and cultured for 5 days in RPMI (Gibco) containing 10% FBS, 0.5% gentamycin, 1% L-glutamine, 50 ng/ml human M‐CSF (eBioscience) for plating. The media was exchanged every 2–3 days until day 5, at which point PBMCs‐derived macrophages were used for *in vitro* experiments.

Human adult cardiac fibroblasts. Human Cardiac Fibroblasts (HCF, adult, Sigma-Aldrich, 306-05A) were kept in culture with Cardiac Fibroblast Growth Medium (Sigma-Aldrich, 316-500). The media was exchanged every 2–3 days until the cells reach 70% confluency. Cells were used for the experiments at passage 2.

**Cell Culture**

**CDC Cell culture.** CDCs were prepared as described^16^. Briefly, atria and ventricular septum were obtained from the healthy hearts of deceased tissue donors. Tissue was chopped, mixed in 1:4 atria to septum ratio, washed, and seeded on CellBIND flasks (Corning). Explants were incubated at 37°C, 5% CO_2_, and 5% O_2_ in IMDM media supplemented with 20% FBS, for 2 – 3 weeks until outgrowth reached 80% confluence. Cells were then harvested using TrypLE Select (Thermo Fisher), filtered through a 100 µm Steriflip unit (Millipore) to remove explants, and resuspended in CryoStore CS10 (Stemcell Technologies) before freezing in liquid nitrogen. When needed, the frozen vial was removed from liquid nitrogen and seeded on Ultra-Low Attachment flasks to form cardiospheres. CDCs were formed by seeding cardiospheres on fibronectin-coated flasks and culturing at 37°C, 5% CO_2_, and 5% O_2_ in IMDM supplemented with 10% FBS. Cells were conditioned at passage 5 or subjected to a second cardiosphere step and conditioned 2 passages after culturing on fibronectin-coated plates.

**Immortalized CDC derivation and culture.** CDCs were derived as described^16^. To make CDCs potent, the desired amount of shPeg1 (Santa Cruz Biotechnology, Cat # sc-61315-V) and SV40 T+t (Applied Biological Materials, Cat # LV614) Lentiviral Activation Particles were applied to the attached CDCs to achieve an MOI of 20.^2^ After 24 hrs transduction, virus was removed, and fresh complete media was added for cell recovery for a further 24 hrs. Cells were then subjected to selection media until the cell line was established**.**

***In vitro* experiments**

**Bromodeoxyuridine (BrdU) Assay.** Macrophages were isolated from rat femurs as previously described and plated in 8-chamber slides at a density of 1x10^6^ cells/ml in complete medium (IMDM + 10% FBS + human recombinant M-CSF 20ng/mL). On day 5 complete medium was removed and replaced with IMDM 2% FBS for all test conditions. Cells were exposed to PBS, 1:100 IMEX (cells:EVs), 80nM yREX3, or scrambled-sequence, together with 10 µM BrdU labeling solution and incubated for 24 hours at 37ºC in a CO_2_ incubator. After 24 hours cells were washed twice in PBS for about 5 seconds per wash, and then three more times with PBS for two minutes each. After fixing in PFA 4% for 10 minutes and permeabilizing, cells were incubated in 2.5M HCL for 30 minutes at room temperature. HCl was then neutralized with 0.1M sodium borate buffer pH 8.5 for 30 minutes at room temperature and washed three times in PBS. Immunostaining was then performed using an anti-BrdU primary antibody [BU1/75 (ICR1)], anti-CD68 primary antibody (Abcam ab125212), Goat Anti-Rat IgG H&L (Alexa Fluor® 488) (ab150157), Goat Anti-Rabbit IgG H&L (Alexa Fluor® 594) (ab150080).

**Migration assay.** Bone marrow-derived monocytes were seeded onto 6-well plates and differentiated into mature macrophages using IMDM + 10% FBS + 20ng/mL human recombinant M-CSF (eBioscience). On day 5 macrophages were exposed to PBS, IMEX, yREX3, or scrambled-sequence for 24 hours in IMDM 2% FBS. The day after, macrophages were lifted using 2 ml of CTS™ Versene™ Solution (Gibco™). Cells were counted and re-seeded onto 5.0-µM pore size transwells (Costar) in serum-free IMDM. At the bottom of the plate, IMDM supplemented with 10% FBS was used to create the gradient, and cells were incubated overnight. The following day cells were gently removed from the upper side of the transwell using a cotton swab. The underside of the transwell was stained for 20 min at RT using Crystal Violet solution. After staining, cells were gently washed several times with water until the wash ran clear. Three images were captured of each transwell at 20x magnification (three per condition). Quantification of cell migration was done using ImageJ. To test whether yREX3 signaling is active in more clinically-relevant samples than those from rats, human macrophages were also studied (see **Supplementary online materials** for details).

**CCK-8 Assay.** Bone marrow-derived monocytes were seeded onto 48-well plates and differentiated into mature macrophages using IMDM + 10% FBS + 20 ng/mL human recombinant M-CSF (eBioscience). On day 5 macrophages were exposed to PBS, IMEX, yREX3, or scrambled-sequence for 8-24 hours in IMDM 2% FBS. The day after, the medium was replaced, 30 µl of WST-8 solution (Cell Counting Kit 8 ab228554) was added in 300 µl of IMDM 2% FBS, corresponding to a final volume of 330 µl/well (48-well plate), and cells incubated for 30 minutes at 37ºC. The absorbance increase was measured at time zero (blank) and after 3-4 hours at 460 nm. The blank was subtracted for every well.

Additional detailed Methods appear in the online Supplement.

**EV preparation and isolation.** Primary CDCs were conditioned in Iscove’s Modified Dulbecco’s Medium (IMDM) without supplementation for 15 days at 37°C and 20% O_2_. Immortalized CDCs were grown to confluence at 20% O_2_ at 37°C, and then cells were serum-free at 2% O_2_ at 37°C overnight after three washes. Conditioned media was collected and filtered through a 0.45 μm filter to remove apoptotic bodies and cellular debris and EVs were purified using centrifugal ultrafiltration with a 1000 KDa Centricon Plus-70 Centrifugal Filter (Millipore).

**EV characterization**

**Nanosight.** Particle size and concentration were measured using NanoSight NS300 (Malvern). The parameters for Nanosight acquisition and analysis were as follows:

camera levels: 14; detection Threshold: 5; videos were taken per sample: 3-4; video duration: 30 sec. Non-EV fraction and non-conditioned culture media were also analyzed using the same parameters.

**Cryo-Transmission electron microscopy.**

Cryo-transmission electron microscopy of IMEX was performed commercially using Creative Biostructure (Shirley, NY). Briefly, a 5 μL aliquot was placed on a thin copper grid (Quantifoil) that had been glow discharged. For preparation of the grid, the sample was loaded into the freezing chamber at a low temperature (0-5°C) under humidity control (100%). After blotting for 3 sec with filter paper, the specimen was rapidly frozen with cryogen, liquid ethane cooled by liquid nitrogen. The prepared grid was mounted on a 200kV FEI Tecnai F20 electron microscope.

**Number of EVs/µg of proteins.** Different concentrations of IMEX were resuspended in PBS and protein concentration was measured using a DC Protein Assay kit (Bio-Rad). The numbers of EVs/µg of proteins were plotted together.

**IMEX.** For *in vitro* experiments, cells were exposed to IMEX in a 1:100 ratio (cells to EVs) in IMDM medium supplemented with 2% FBS at day 5.

**yREX3 mimics.** Macrophages were transfected with 80nM Y RNA4-derived fragment, yREX3 (5’- CCCCCCACTGCTAAATTTGACTGGTT-3’, synthesized by Integrated DNA Technologies) or scrambled control (5’-GAUCCCUCAUGUGCACCGUUCAUCAU-3’, synthesized by Integrated DNA Technologies) using DharmaFECT (Horizon) to generate Mφ^yREX3^ and Mφ^Scr^. Transfections were performed on day 5 in IMDM medium supplemented with 2% FBS for 24, 48, or 72 hours. Two mutants wherein the six-cytosine leader sequence was substituted with purine stretches of either guanine (yREX3^s6G^) or adenine (yREX3^s6A^) were also synthesized.

**siPick1 transfection.** Macrophages were seeded in 10cm dishes or 6-well plates and on day 5 they were transfected with Pick1 rat siRNA Oligo Duplex (Origene #SR501054) or control sequence. Briefly, siRNA duplexes were reconstituted in 100μl of RNase-free duplex buffer for a final concentration of 20μM, vortexed, and heated to 94°C for 2 minutes. Fresh medium was replaced 30-60 minutes before transfection and a final concentration of 50nM was used to transfect the cells using 1x Transfection buffer and siTran 2.0 reagent to generate Mφ^siPick1^ and Mφ^siScramble^.

**siPTBP3 transfection.** Macrophages were seeded in a 6-well plate and on day 5 they were transfected with PTBP3 Rat siRNA Oligo Duplex (Origene #SR507192) or control sequence. Briefly, siRNA duplexes were reconstituted in 100μl of RNase-free duplex buffer for a final concentration of 20 μM, vortexed, and heated to 94°C for 2 minutes. Fresh medium was replaced 30-60 minutes before transfection and a final concentration of 50nM was used to transfect the cells using 1x Transfection buffer and siTran 2.0 reagent to generate Mφ^siPTBP3^ and Mφ^siScr^.

**RG108.** Macrophages were exposed to vehicle or to yREX3 and scrambled-sequence for 48 hours, in the presence or not of the DNA Methyltransferase Inhibitor (Calbiochem, #CAS 32675-71-1) at a final concentration of 100µM. Cells were collected, and RNA or DNA isolation was performed.

**Pick1 overexpression.** Macrophages were seeded in 10cm dishes or 6-well plates and on day 5 they were transfected with Pick1 Rat Tagged ORF Clone (Origene #RR201340). Briefly, the plasmid was resuspended in 100µl of sterile water to dissolve the DNA. FuGene HD (Promega, E2311) was used to transfect the cells in a 6:1 ratio (FuGene:DNA) using an 830ng DNA/10cm dish in IMDM 10% FBS for 24-48 hours.

**RNA-Protein pull down.** A single desthiobiotinylated cytidine bisphosphate to the 3´ end of the RNA strands was attached using T4 RNA ligase (Thermo Scientific Pierce RNA 3´ End Desthiobiotinylation Kit). yREX3, scramble, control, and piREX^s6A^ RNAs were labeled using desthiobiotinylated cytidine bisphosphate using the kit procedure. Labeled RNA was captured using 50µL of streptavidin magnetic beads in RNA Capture Buffer for 30 minutes at room temperature. Beads were washed twice in 20mM Tris (pH 7.5), once in Protein-RNA Binding Buffer, and 200-400µg of BMDM macrophage extract was added. Samples were incubated for 1-2 hours at 4°C, washed three times with Wash Buffer, and eluted after 15 minutes of incubation at 37°C with Biotin Elution Buffer. Samples were either used for mass-spectrometry analysis or ELISA (PTBP3 ELISA).

**PTBP3 ELISA.** Samples obtained from RNA-Protein pull-down were analyzed for PTBP3 expression using rat PTBP3 Elisa kit (Abbexa, abx540609) according to the manufacturer’s protocol.

**PTBP3 immunoprecipitation.** Mφ^yREX3^ and Mφ^Scramble^ were lysed in IP-MS Cell Lysis Buffer and then 1000µg of lysates were incubated overnight at 4°C with 5µg of PTBP3 IP antibody (Santa Cruz Biotechnology, sc-398105) to form the immune complex. The antigen sample/antibody mixture was incubated for 1 hour with Pierce protein A/G magnetic beads. Beads were then washed with wash buffers A and B and eluted after 10 minutes of incubation at room temperature with 100µl of Elution Buffer. Samples were used for ELISA (RAVER1 ELISA) or qPCR for yREX3.

**RAVER1 ELISA.** Samples obtained from Immunoprecipitation were analyzed for RAVER1 expression using rat RAVER1 ELISA kit (Abbexa, abx541456) according to the manufacturer’s protocol.

**Supplementary Figure Legends**

**Supplementary Figure 1: RNA distribution in immortalized CDCs and primary CDCs and their EVs.** (A) Next-generation sequencing of imCDCs and pCDCs, and (B) IMEX and CDC-EVs, identified a diversity of RNA species including micro-RNA (miRNA), hairpin, PIWI RNA (piRNA), ribosomal RNA (rRNA), transfer RNA (tRNA), messenger RNA (mRNA) and other RNA. (C) Northern Blot analysis of yREX3 RNA expressed by ImCDCs and IMEX (on the left the gel stained with SYBR Green before transfer, and membrane after transfer with 1 second exposure to visualize ladders, and 2 minutes exposure to visualize bands on the samples).

**Supplementary Figure 2: yREX3 induces significant transcriptomic changes in bone marrow-derived macrophages.** (A) Heat-maps for transcriptomic data of cardiomyocytes (CM), cardiac fibroblasts (CF) and BMDM (Mφ) exposed to yREX3 (80nM for 24 hrs, purple box) show significant differential gene expression compared to vehicle-exposed cells (gold box, n=3 samples/group). Gene Ontology analysis of top pathways and top up- and down-regulated genes in cardiomyocytes (CM, B, C), cardiac fibroblasts (CF, D, E), and macrophages (Mφ, F, G; n=3 biological replicates per group). (H) Efferocytosis markers identified in BMDM (Mφ) exposed to yREX3 (80nM for 24 hrs ) compared to vehicle-treated BMDM. (I) QPCR quantification of isolated peripheral blood mononuclear cells from animals intravenously infused with 400 ng of yREX3 or vehicle control (n=3 rats/group). Analysis was done using Student’s independent t-test * = p < 0.05, ** = p < 0.01 and *** = p < 0.001.

**Supplementary Figure 3: yREX3 methylates pick1 in macrophages.** (A) Western blot for cytoplasmic and nuclear fractions in BMDM transfected with yREX3, scramble and vehicle at 24hrs showing enrichment of histone proteins (H3A) in the nuclear fractions and pan actin in the cytoplasmic fractions. (B) Analysis of global methylation levels in BMDM 24 hours post-exposure to vehicle, yREX3 or Scramble. (C) Analysis of global methylation levels in BMDM 72 hours post-exposure to vehicle, yREX3 or Scramble. (D) Data from Whole Genome Bisulfite Methylation showing hyper- or hypo-methylated genes in BMDM exposed to yREX3 compared to vehicle (q values from data at 24 hours and 48 hours ). (E) Schematic of the experimental design. Targeted methylation assays were designed to interrogate the DNA methylation status of 56 CpG sites in the 5’ Upstream to Intron 6 regions of the Rat Pick1 gene. (F) Pick1 suppression by yREX3 was not observed in cardiomyocytes (CM, G),cardiac fibroblasts and (H) human cardiac fibroblasts. (I) Analysis of global methylation levels in BMDM 24 hours post-exposure to vehicle, yREX3 or siPick1. (J) (A) Mass-spectrometry analysis of RNA-Protein pull-down showing peptide hits identified in yREX3 and scrambled-exposed macrophages of DNA Methyltransferases 3a, 3b and 3-like. (B, C, F- H) All data were presented as mean ± SEM, and comparison between groups was evaluated using a Student’s independent t-test and one-way ANOVA with Tukey’s post-test with * = p < 0.05, ** = p < 0.01, and *** = p < 0.001.

**Supplementary Figure 4: yREX3 modulates Smad signaling.** (A, B) Phosphorylation levels of Smad1 and Smad2 expressed as phosphorylated/total in vehicle- and yREX3-exposed BMDM (n=5-6 technical replicates/group). (C) QPCR for TGFβ-1 expression in vehicle, scramble and yREX3-exposed BMDM at 24 hours (n=3-4 biological replicates/group, data presented as fold change compared to Vehicle). (D) Secretion of TGFβ-1 in vehicle, scramble, and yREX3-exposed BMDMs at 24 and 48 hrs (n=3 biological replicates/group). (E) Expression levels of PKCε and PKCα and their phosphorylation levels in vehicle- and yREX3-exposed BMDM (n=5-6 technical replicates/group). All data presented as mean ± SEM, comparison between groups using Student’s independent t-test or one-way ANOVA with Tukey’s post-test with * = p < 0.05, ** = p < 0.01 and *** = p < 0.001.

**Supplementary Figure 5: Pick1 suppression recapitulates yREX3 signaling in macrophages**. (A) Confirmation of Pick1 suppression using a siRNA against rat pick1 in qPCR compared to vehicle and a silencing scramble. (B) Proliferation rates calculated at 24 hours in BMDM exposed to vehicle, siScramble, and siPick1 using a colorimetric CCK-8 assay (n=6 replicates from two different experiments). (C) Confirmation of pick1 overexpression in BMDM using an overexpressing vector for rat pick1 in protein levels at 24 hours compared to vehicle and (D) representative blots; pan-actin was used to normalize the data. (E) Pick1 overexpression validation in qPCR at the same time-point. (F) Proliferation rates calculated at 24 hours in BMDM exposed to vehicle, and Pick1 OE. (G, H) yREX3 potentiates human-derived macrophage migration at 24 hours post-exposure using a Bowden chamber assay (migration calculated as integrated density/area, n=2 independent experiments) and representative images (Scale bar: 100µm). (I, J) Efferocytosis assay showing increased uptake of DiO-labeled dead rat cardiomyocytes by yREX3- exposed human-derived macrophages (representative images taken at 48 hours, n=5-6 replicates/group from 2 independent experiments, Scale bar: 200µm).All data are presented as mean ± SEM, comparisons were analyzed using Student’s independent t-test or one-way ANOVA with Tukey’s post-test with * = p < 0.05, ** = p < 0.01, and *** = p < 0.001; scale bars: 200µm.

**Supplementary Figure 6: yREX3 activates pSmad3 in infarct area.** (A) Merged immunohistochemistry images of entire heart sections for CD68 (red), Smad3 phospho-Ser213 (green), and DAPI. (B) Quantification of immunohistochemistry for CD68+ macrophage infiltration in the heart (n=4 animals/group). (C) Quantification of Smad3 phospho-Ser 213 in the heart (n=4 animals/group). (D) Merged immunohistochemistry images of heart sections for CD68 (red), Smad3 phospho-Ser213 (green), and DAPI; Vimentin (green), Smad3 phospho-Ser213 (red), and DAPI; alpha-sarcomeric actin (red), Smad3 phospho-Ser213 (green), and DAPI. Scale bars: 100µm.

**Supplementary Figure 7: PTBP3/Raver1 genes are differentially expressed between bone marrow-derived macrophages (BMDMs), cardiac fibroblasts (CFs), and neonatal rat ventricular myocytes (NRVMs). (**A-C) Gene expression for PTBP3, RAVER1, and RAVER2 expression levels in vehicle- and yREX3-exposed BMDM, cardiac fibroblasts (CFs), and neonatal cardiomyocytes (NRVMs) with yREX3 exposure at 24 hours (data presented as fold change compared to BMDM). All data are presented as mean ± SEM.

**
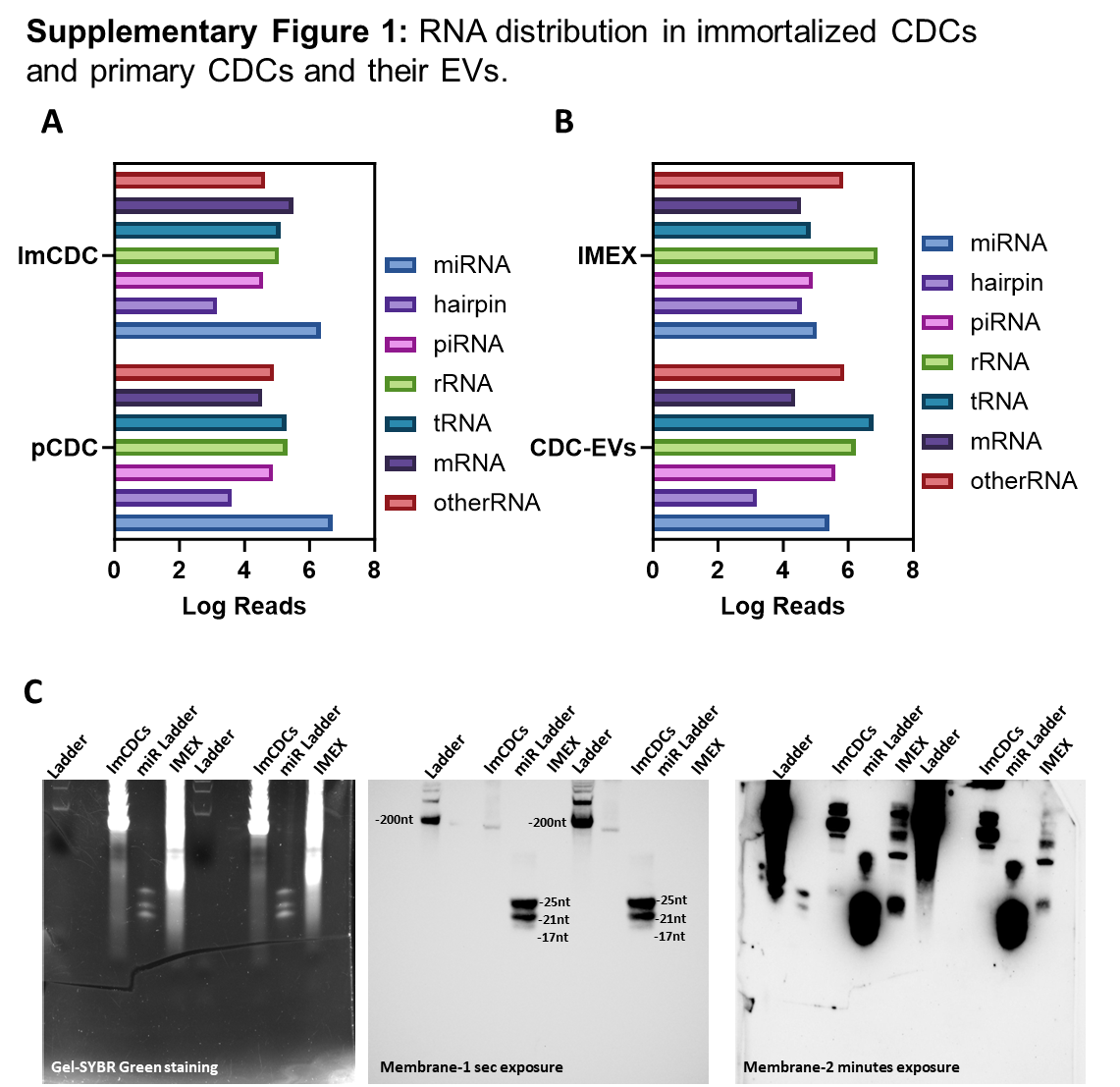
**

**
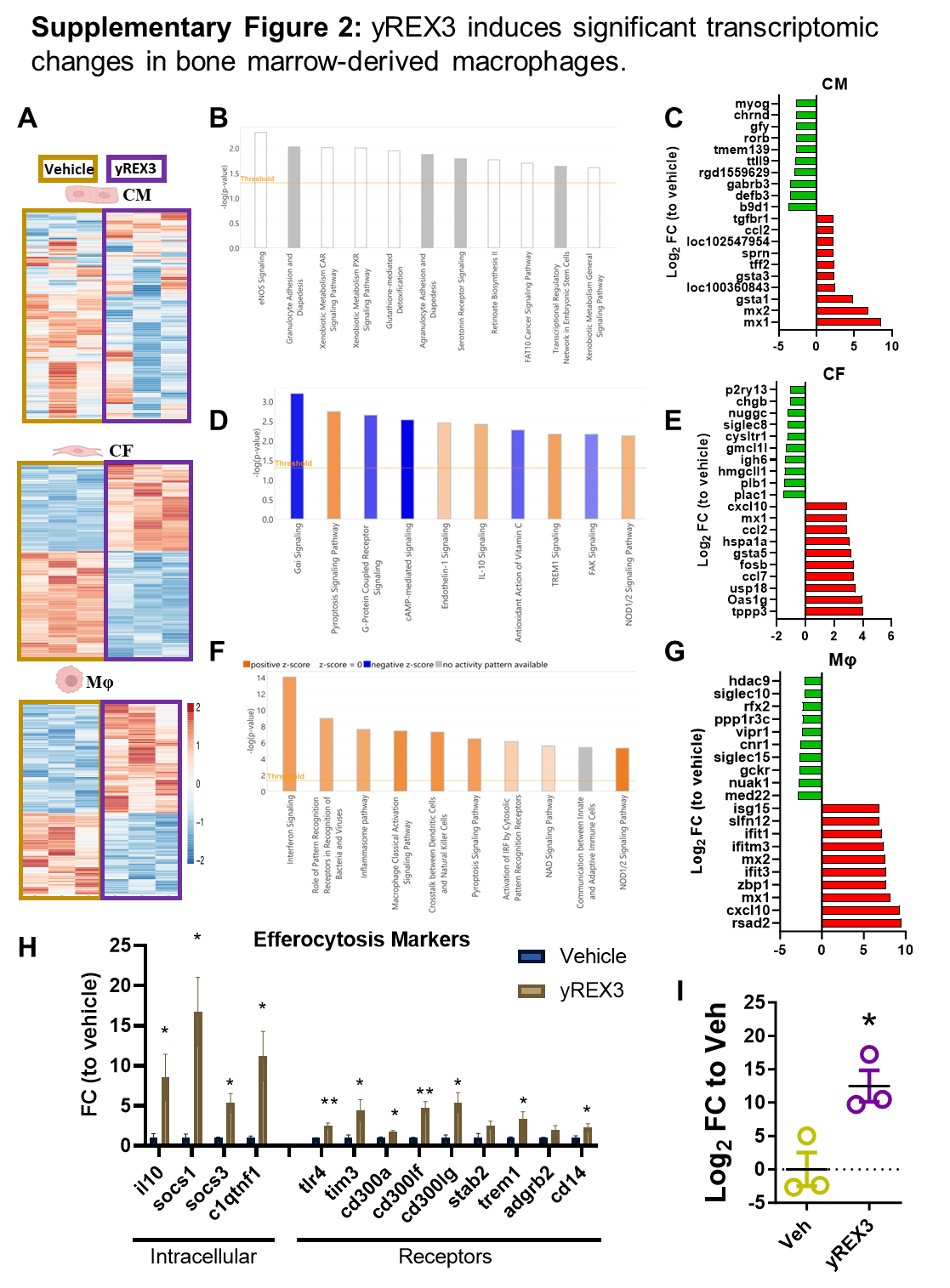
**

**
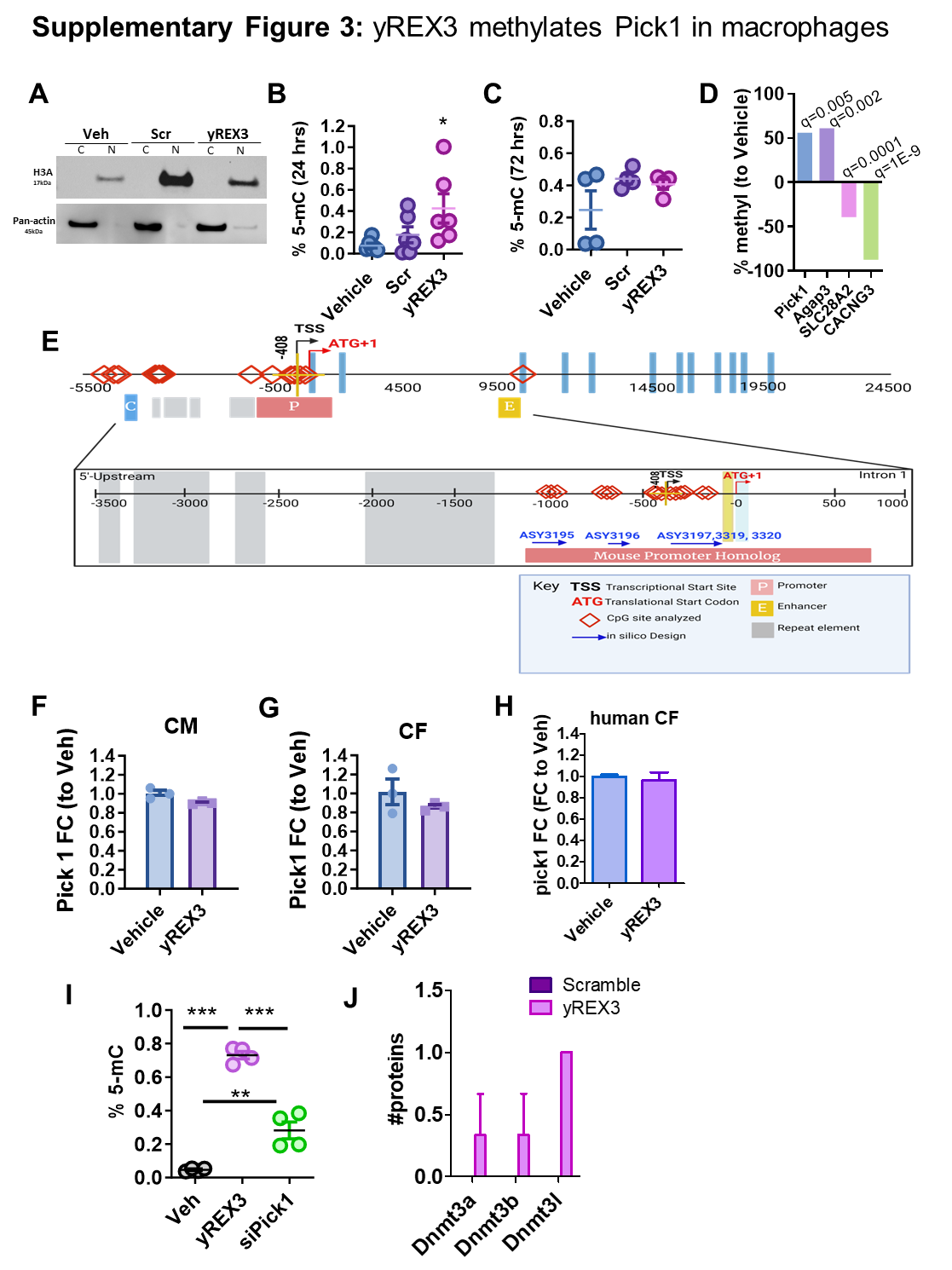
**

**
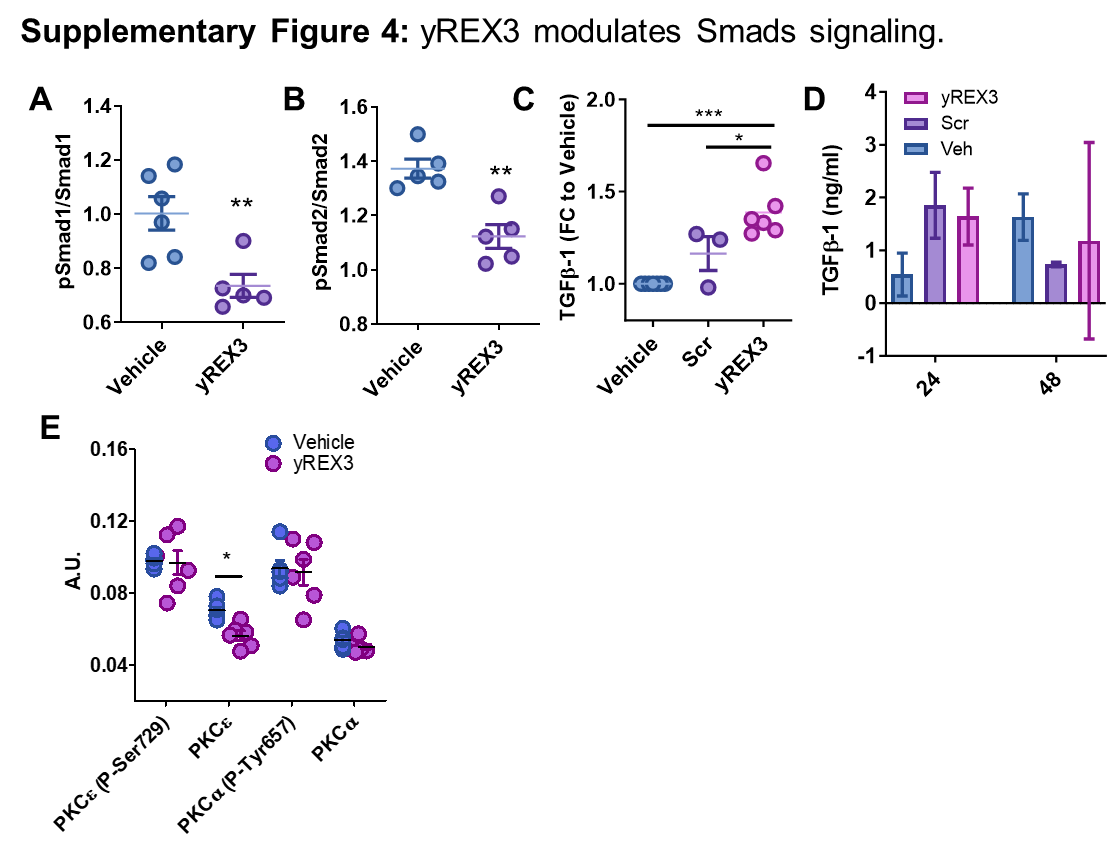
**

**
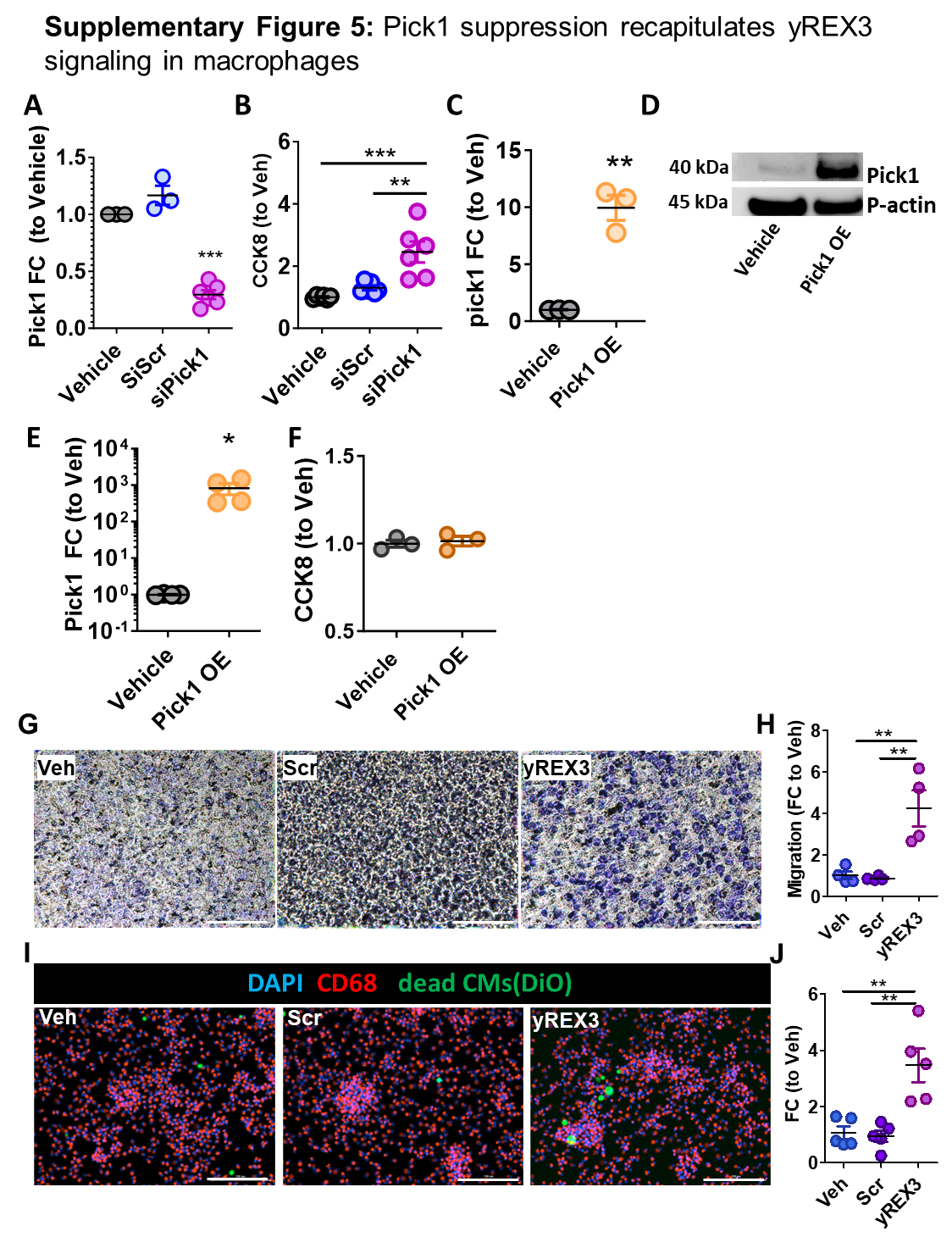
**

**
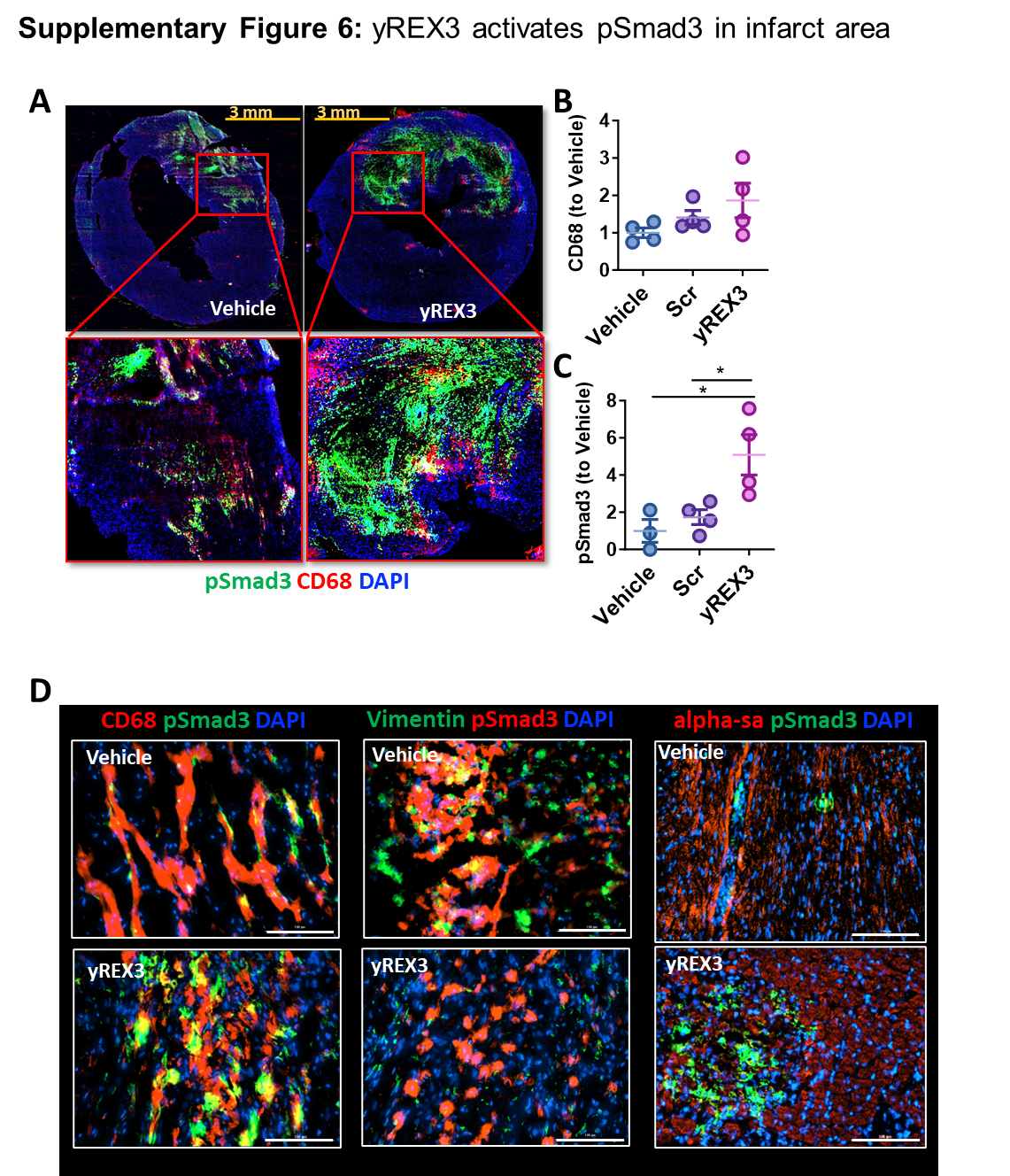
**

**
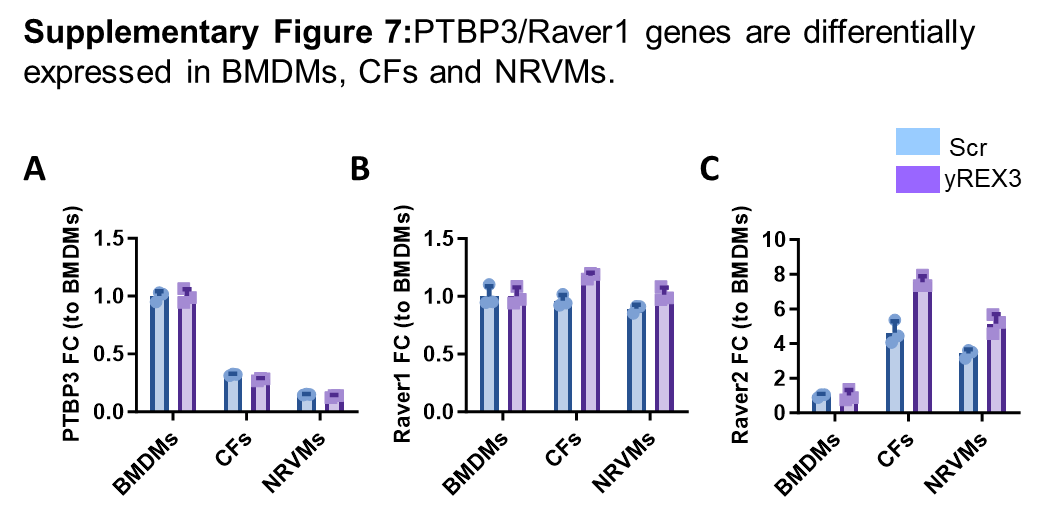
**
